# Nuclear actin-dependent *Meg3* expression suppresses metabolic genes by affecting the chromatin architecture at sites of elevated H3K27 acetylation levels

**DOI:** 10.1093/nar/gkaf280

**Published:** 2025-04-14

**Authors:** Nadine Hosny El Said, Wael Abdrabou, Syed Raza Mahmood, Tomas Venit, Youssef Idaghdour, Piergiorgio Percipalle

**Affiliations:** Program in Biology, Division of Science and Mathematics, New York University Abu Dhabi (NYUAD), PO Box 129188, Abu Dhabi, United Arab Emirates; Center for Genomics and Systems Biology, New York University Abu Dhabi (NYUAD), PO Box 129188, Abu Dhabi, United Arab Emirates; Center for Genomics and Systems Biology, New York University Abu Dhabi (NYUAD), PO Box 129188, Abu Dhabi, United Arab Emirates; Program in Biology, Division of Science and Mathematics, New York University Abu Dhabi (NYUAD), PO Box 129188, Abu Dhabi, United Arab Emirates; Program in Biology, Division of Science and Mathematics, New York University Abu Dhabi (NYUAD), PO Box 129188, Abu Dhabi, United Arab Emirates; Center for Genomics and Systems Biology, New York University Abu Dhabi (NYUAD), PO Box 129188, Abu Dhabi, United Arab Emirates; Program in Biology, Division of Science and Mathematics, New York University Abu Dhabi (NYUAD), PO Box 129188, Abu Dhabi, United Arab Emirates; Center for Genomics and Systems Biology, New York University Abu Dhabi (NYUAD), PO Box 129188, Abu Dhabi, United Arab Emirates

## Abstract

Nuclear actin mediates enhancer-dependent transcriptional regulation at compartment level, playing critical roles in 3D genome organization. In β-actin depleted cells, H3K27 acetylation is enhanced, directly affecting enhancer-dependent transcriptional regulation and gene expression changes during compartment-switching. Here, we report these mechanisms are influenced by the long non-coding RNA (lncRNA) *Meg3*. Bulk RNA-seq analysis and qPCR on wild-type (WT), heterozygous (HET), and β-actin knockout (KO) mouse embryonic fibroblasts (MEFs) show that β-actin depletion significantly alters expression of several lncRNAs, including *Meg3*. Results from ChIRP-seq, ChIRP-MS, and fRIP-qPCR revealed that in β-actin KO cells, Meg3 becomes enriched and binds to H3K27 acetylation marks within gene regulatory regions. By integrating RNA-seq, H3K27 acetylation ChIP-seq, ATAC-seq, and HiC-seq data through activity by contact (ABC) analysis, we discovered *Meg3* binding disrupts promoter–enhancer interactions in β-actin KO cells. These results, combined with metabolomics in WT, HET, and β-actin KO MEFs, show *Meg3* binding to regulatory regions at sites of increased H3K27 acetylation impairs the expression of genes involved in the synthesis of chondroitin, heparan, dermatan sulfate, and phospholipases. We propose that in β-actin KO cells *Meg3* binds to H3K27 acetylation levels. This interferes with promoter–enhancer interactions, disrupts genome organization, and downregulates gene expression and key metabolic pathways.

## Graphical Abstract

**Graphical Abstract:**
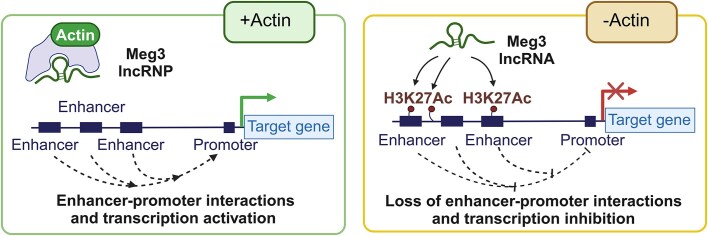

## Introduction

The mammalian genome is hierarchically organized into large chromatin compartments, stretches of either active (A compartment) or inactive chromatin (B compartment), which consist of self-interacting genomic regions known as topologically associating domains (TADs) and chromatin loops. This complex architecture arises from both short- and long-range interactions between intra- and inter-chromosomal loci, including promoter–enhancer contacts, which stabilize chromatin loops and can result in gene activation or repression [1, 2]. Several factors maintain genomic architecture, including CTCF, the cohesin complex [3–7] and nuclear actin, which has recently emerged as a critical regulator of 3D genome organization at compartment level [8–11]. Notably, loss of β-actin leads to significant compartment switching, where chromatin compartments shift between interactions with euchromatin and heterochromatin (A–B or B–A changes). These alterations induce reversible changes in 3D genome architecture and impact both chromatin remodeling activities and nuclear compartmentalization. Indeed, upon β-actin depletion the ATP-dependent SWI/SNF (SWItch/Sucrose Non-Fermentable)/BAF (mammalian SWI/SNF complex) chromatin remodeling complex is impaired and this affects local chromatin accessibility and transcription. In β-actin KO mouse embryonic fibroblasts (MEFs), the Polycomb Repressive Complex 2 (PRC2) replaces SWI/SNF, leading to increased methylation, chromatin compaction, altogether resulting in disrupted enhancer-dependent transcriptional regulation [10, 12–15]. Actin also associates with the heterogeneous nuclear ribonucleoprotein hnRNP U to promote clustering of the RNA polymerase II machinery [16, 17] while at transcription start sites, in complex with the hyperphosphorylated RNA polymerase II C-terminal domain (CTD), it also mediates recruitment of histone-modifying enzymes and enhance RNA polymerase II-mediated transcription elongation [17, 18]. Notably, actin is also co-transcriptionally associated with the nascent ribonucleoprotein complex (RNP) and remains associated with mature RNPs in complex with A/B type hnRNP proteins [19–21] to promote RNP stability [8, 22]. Altogether, these actin-dependent mechanisms profoundly influence gene expression and cellular processes, such as neurogenesis [23], osteogenesis [24], and adipogenesis [25]. In rat peritoneal mast cells, a decrease in ATP leads to elevated nuclear actin levels, indicating a potential role for nuclear actin in cellular stress responses [26]. Similarly, an increase in nuclear actin levels has been observed in monocytic HL-60 cells following stimulation with phorbol ester, suggesting its involvement in regulating macrophage differentiation [27]. Additionally, there is evidence of physiological signals that raise cAMP levels inducing a rapid rise in nuclear actin monomer levels in vascular smooth muscle cells (VSMCs) [26]. In all these cases, β-actin deletion affects cell fate commitment and, therefore, impairment of these actin-based mechanism may be a potential cause of major pathologies.

Chromatin-modifying enzymes are regulated by long non-coding RNAs (lncRNAs) and this critically impacts on various cellular processes such as cell proliferation, differentiation, and, more generally, on the establishment of cell identity [28]. lncRNAs bind to chromatin modifying enzymes and function as guides to anchor them to specific genomic locations or as decoy to sequester them from specific genomic sites, affecting covalent histone modifications. lncRNAs can also affect chromatin accessibility as part of ATP-dependent chromatin remodeling complexes where they serve as scaffold to assemble the complex itself for localized chromatin modification. For instance, the Mhrt lncRNA binds to Brg1 which leads to Brg1 sequestration from genomic DNA loci and inhibits Brg1-dependent gene regulation. The lncRNA *Meg3* (maternally expressed imprinted lncRNA Gene), on the other hand, regulates PRC2. *Meg3* facilitates recruitment of the PRC2 subunits JARID2 and EZH2—a polycomb group histone methyltransferase that targets trimethylated histone 3 lysine 27 (H3K27me3)—to the chromatin for histone H3 methylation. Through this mechanism it is directly involved in the epigenetic control of several genes including genes implicated in stemness [28], epithelial-to-mesenchymal transition (EMT) [29], and enhanced TGF-β (transforming growth factor β) pathway genes [30], where EZH2 recruitment and increased *Meg3* levels mediate transcriptional repression induced by TGF-β signaling [29, 30]. In turn, enhanced TGF-β signaling has an overall impact on chromatin accessibility with increased H3K27 acetylation at enhancers [31]. Enhanced TGF-β signaling is also a downstream effect of nuclear β-actin depletion when extensive genetic reprogramming results from genome re-organization and alterations in promoter–enhancer interactions due to increased H3K27 acetylation [12, 13, 32]. However, the functional significance of a possible association of lncRNAs with increased H3K27 acetylation and their impact on genome organization upon β-actin depletion is not known.

We, therefore, set out to investigate if nuclear actin controls lncRNAs expression levels and whether this may have an impact on the spatial organization of the genome and gene expression. To this end, we used β-actin KO MEFs isolated from an embryonic lethal β-actin KO (β-actin KO MEFs) exhibiting an intact and functional cytoplasmic cytoskeleton as a consequence of increased levels of α-smooth muscle actin [32–34]. For comparison, we used wild-type (WT) MEFs as well as MEFs isolated from a heterozygous condition (HET) where only one of the β-actin alleles is ablated [33]. Using a combination of bulk RNAseq and qPCR (real-time quantitative polymerase chain reaction) analyses performed on total RNA isolated from WT MEFs, β-actin KO MEFs and HET MEFs, we demonstrate that expression of several lncRNAs is directly affected by β-actin depletion. *Meg3* expression is particularly upregulated in β-actin KO cells. Results from ChIRPseq, ChIRP-MS, and formaldehyde crosslinking-RNA immunoprecipitation (f-RIP)/qPCR experiments show alterations in *Meg3* genomic association and *Meg3* enrichment at or close to regulatory sites (enhancers and promoters) specifically exhibiting elevated H3K27 acetylation in the KO condition. Results from activity by contact (ABC) analysis using RNA-seq, H3K27ac ChIP-seq, ATAC-seq, and HiC-seq data in WT and β-actin KO MEFs show that *Meg3* enrichment at H3K27ac sites leads to loss of promoter–enhancer interactions. This, in turn, contributes to the repression of genes involved in chondroitin, heparan, dermatan sulfate, and phospholipases biosynthetic pathways. We propose that *Meg3* binds to sites of enhanced H3K27 acetylation. This, in turn, interferes with promoter–enhancers interactions, impairing local genome organization (or DNA looping), and leads to gene expression alterations.

## Materials and methods

### Cell culture

WT, HET, and β-actin KO MEFs are a gift of Dr. Christophe Ampe, University of Gent, Belgium. Cells were maintained and cultured with Dulbecco’s modified Eagle medium (DMEM) with high glucose, 10% fetal bovine serum (FBS), 100 U/ml penicillin, and 100 μg/ml streptomycin, in a humidified incubator with 5% CO_2_ at 37°C.

### ChIP-seq

WT or β-actin KO MEFs were crosslinked using 1% formaldehyde (Sigma cat. No. F8775) for 10 min. Then, quenching was carried out using 0.125 M glycine for 5 min. Cells were then lysed using lysis buffer 1: LB1 (50 mM Hepes KOH, pH 7.5, 10 mM NaCl, 1 mM EDTA, 10% glycerol, 0.5% NP-40, and 0.25% Triton X-100). Nuclei were pelleted, collected, and then washed using lysis buffer 2, LB2, containing 10 mM Tris–HCl, pH 8, 200 mM NaCl, 1 mM EDTA, and 0.5 mM ethylene glycol bis(2-aminoethyl ether)-N,N,N',N'-tetraacetic acid (EGTA). This was followed by lysis using lysis buffer 3, LB3, containing 10 mM Tris–HCl, pH 8, 100 mM NaCl, 1 mM EDTA, 0.5 mM EGTA, 0.1% Na-deoxycholate, and 0.5% *N*-laurylsarcosine. Chromatin was then sheared using Qsonica Sonicator (4 cycles of 3 min at 70% amplitude), and then checked on 0.8% agarose gel. Hundred micrograms of fragmented chromatin was mixed with 5 g of *Ezh2* (D2C9) XP Rabbit mAb antibody, Cell signaling. The protein–antibody immunocomplexes were recovered by the Pierce Protein A/G Magnetic Beads (Thermo Scientific). Beads and attached immunocomplexes were washed twice using low salt wash buffer (LS) (0.1% SDS, 2 mM EDTA, 1% Triton X-100, 20 mM Tris–HCl pH 8, 150 mM NaCl), and high salt (HS) wash buffer (0.1% SDS, 2 mM EDTA, 1% Triton X-100, 20 mM Tris–HCl pH 8, 500 mM NaCl), respectively. The beads were then resuspended in elution buffer containing 50 mM Tris–HCl pH 8, 10 mM EDTA, 1% Sodium Dodecyl Sulfate (SDS). De-crosslinking was achieved through adding 8 μl of 5 M NaCl and incubating at 65°C overnight. RNase A (1 μl, 10 mg/ml) was added to the tube for a 30 min incubation at 37°C. Then, 4 μl of 0.5 M EDTA, 8 μl of 1 M Tris–HCl, and 1 μl of 20 mg/ml proteinase K (0.2 mg/ml) were added for a 2 h incubation at 42°C to digest the chromatin. DNA was then purified by QIAquick PCR purification kit (Qiagen, Germantown, MD, USA) for qPCR analysis (for primers see Supplementary Table S4) and sequencing. ChIP-seq library preparation was done using the TruSeq Nano DNA Library Prep Kit (Illumina, San Diego, CA, USA) and then sequenced with the HiSEq 2500 sequencing platform (performed at the NYUAD Sequencing Center).

### ChIRP-seq

Biotin-labeled antisense oligo probes (see Supplementary Table S4) were purchased from Integrated DNA Technologies (IDT) and were designed against Mouse *Meg3* lncRNA according to Chu *et al.* [35] using the following criteria: 1 probe per 100 bp of length, GC% target = 45, oligonucleotide length of 20, and a spacing length of 6080 bp. Probes, numbered and divided into even and odd numbered pools, were diluted up to 100 μM concentrations. WT and β-actin KO MEFs were fixed using 1% glutaraldehyde for 10 min, and then quenched using one-tenth volume of the 1.25 M glycine for 5 min. The cells were then pelleted, lysed, and sonicated using a Qsonica Sonicator under a 30 s on, 30 s off cycle for 3–4 h (four cycles of 3 min at 70% amplitude), until a clear lysate was obtained. The sonicated chromatin was then centrifuged at 16 100 RCF (Relative Centrifugal Force) for 10 min at 4°C and aliquoted into 1 ml of samples. RNA and DNA INPUT were withdrawn from the samples and stored at −20°C. Each 1 ml of chromatin sample was mixed with 2 ml of hybridization buffer, followed by the addition of 1 μl of 100 pmol/μl probes per 1 ml of chromatin. The samples were then incubated at 37°C for 4 h with shaking. For the pulldown assay, streptavidin-conjugated C-1 magnetic beads were washed with lysis buffer before use. Hundred microliters of beads per 100 pmol of probes were added to each sample and incubated for 30 min at 37°C with shaking. After five rounds of washing, the beads were resuspended in 1 ml of wash buffer. From each 1 ml of sample, 100 μl was used for RNA isolation using Trizol, while the remaining fraction was used for DNA isolation and followed by library preparation and sequencing. The efficiency of the Meg3 lncRNA pulldown was assessed using qPCR, with NONO serving as the negative control (Miao *et al.*, 2018).

### ChIRP-MS

The ChIRP-MS protocol was adapted from the Chang lab [36, 37]. Briefly, 20 p150 flasks of either WT or β-actin KO MEFs were crosslinked for 30 min with 3% formaldehyde and quenched with 0.125 M glycine for 5 min. For a total of ∼500 million cells, 100 mg of cell pellet, centrifuged at 2000 RCF as advised, was resuspended well in 1 ml of lysis buffer. Cells were sonicated using the Qsonica Sonicator until lysis got clear, and then DNA was extracted and run to check that the sonication was enough to proceed.

### ATAC-seq

The ATAC-seq data were obtained from Mahmood *et al.* [13]. Briefly, two biological replicates were used for each condition. Samples with 50 000 cells per condition were shipped in frozen medium including DMEM (Dulbecco's Modified Eagle Medium) with 50% FBS (fetal bovine serum) and 10% Dimethyl Sulfoxide (DMSO) on dry ice to Novogene (Beijing, China). All subsequent processing was performed by Novogene using standard DNA extraction and library preparation protocols. Cell nuclei were isolated, mixed with Tn5 Transposase with two adapters, and tagmentation was performed for 30 min at 37°C. The fragmented DNA was purified and amplified with a limited PCR cycle using index primers. Libraries were prepared according to recommended Illumina NovaSeq6000 protocols. All ATAC-Seq processing was performed by Novogene (Beijing, China).

### HiC-seq

The HiC-seq data sets were from Mahmood *et al.* [13]. Briefly, two biological replicates were used for each condition. Samples with 1 million cells per condition were fixed with 2% formaldehyde for 10 min. The cell pellets were washed twice by 1× phosphate-buffered saline (PBS) and then stored at −80°C. Frozen pellets were shipped on dry ice to Genome Technology Center at NYU Langone Health, NY. All subsequent processing was performed by Genome Technology Center at NYU Langone Health using standard DNA extraction and library preparation protocols. Hi-C was performed at Genome Technology Center at NYU Langone Health, NY from 1 million cells. Experiments were performed in duplicates following the instructions from Arima Hi-C kit (Arima Genomics, San Diego, CA). Subsequently, Illumina-compatible sequencing libraries were prepared by using a modified version of KAPA HyperPrep library kit (KAPA BioSystems, Willmington, MA). Quality check steps were performed to assess the fraction of proximally ligated DNA labeled with biotin, and the optimal number of PCR reactions needed to make libraries. The libraries were loaded into an Illumina flowcell (Illumina, San Diego, CA) on a NovaSeq instrument for paired-end 50 reads.

### ATAC-seq and ChIP-seq (*Ezh2*) preprocessing

Raw reads were quality trimmed using Trimmomatic 64 and analyzed with FastQC (http://www.bioinformatics.babraham.ac.uk/projects/fastqc) to trim low-quality bases, systematic base calling errors, and sequencing adapter contamination. Specific parameters used were “trimmomatic_adapter.fa:2:30:10 TRAILING:3 LEADING:3 SLIDINGWINDOW:4:15 MINLEN:36.” Surviving paired reads were then aligned against the mouse reference genome (GRCm38) using Burrows-Wheeler Aligner BWA-MEM 65. The resulting BAM alignments were cleaned, sorted, and deduplicated (PCR and optical duplicates) with PICARD tools (http://broadinstitute.github.io/picard). Bigwig files were generated using deeptools 66 command bamCoverage -bs 10 -e –ignoreDuplicates –normalizeUsingRPKM. Encode blacklisted regions were removed and replicate bigwig files were averaged using the deeptools 66 command bigwigCompare –operation mean. Bigwig files were analyzed with computeMatrix function of deeptools 66 to plot average signal around regions of interest.

### ATAC-seq differential analysis

Differential analysis was performed using HOMER 60. Processed bam files were converted to HOMER tag directories followed by annotation and differential analysis with the scripts annotatePeaks.pl and getDiffExpression.pl. ATAC-Seq peaks were called on cleaned, deduplicated bam files using macs2 with the parameters -q 0.05 -g mm –keep-dup all –nomodel –shift − 100 –extsize 200 -B –broad -f BAMPE 67. Peaks common to two replicates in each condition were retained and merged using homer command mergePeaks. Differential peaks were identified and annotated using homer scripts annotatePeaks.pl and getDiffExpression.pl. Peaks showing more than two-fold change were divided into three clusters containing 84, 391, and 539 peaks using deeptools kmeans clustering. Clusters containing 391 and 539 peaks were classified as activated and repressed and used for further analysis. Promoters showing more than twofold change in ATAC signal with FDR (False Discovery Rate) < 0.05 were identified using HOMER command annotatePeaks.pl tss mm10 -size -1000 100 -raw and getDiffExpression.pl. To identify enhancers showing more than two-fold change in ATAC signal with FDR < 0.05, enhancer–promoter pairs for MEFs were downloaded from http://chromosome.sdsc.edu/mouse/download/Ren_supplementary table7.xlsx18. Regions 2 kb upstream and downstream of enhancer peaks were analyzed using annotatePeaks.pl and getDiffExpression.pl. To make plots of nucleosome occupancy, bam files were filtered to remove insert sizes < 30 bp and processed with the NucleoAtac 68 pipeline using default settings and previously called atac-seq peaks. The resulting bedgraph files were converted to bigwigs and nucleosomal signal was plotted in 5 kb regions surrounding TSSs of interest using deeptools.

### *Ezh2* differential analysis

Differential analysis was performed using HOMER. Processed bam files were converted to HOMER tag directories followed by annotation and differential analysis with the scripts annotatePeaks.pl and getDiffExpression.pl. Peaks were called using the macs2 67 command: macs2 callpeak -t Rep-1.bam Rep-2.bam -q 0.05 -g mm -c Input.bam (bam file for the relevant input control) –keep-dup all -B -f BAMPE –nolambda –nomodel –broad. WT and KO peaks were merged using HOMER command mergePeaks. Differential peaks were identified and annotated using homer scripts annotatePeaks.pl and getDiffExpression.pl. Overlap of TSSs with known polycomb targets for Fig. 2D was estimated using data from 19. A transcript was regarded as a target if at least one polycomb subunit bound within 1 kb of its TSS. GC and CpG content for *Ezh2* peaks was obtained using the HOMER command annotatePeaks.pl -CpG

### *Brg1*, H3K9me3, and H3K27me3 ChIP-seq analysis

For *Brg1*, H3K9me3, and H3K27me3 analysis, bigwig files were downloaded from GEO accession number GSE100096. Blacklisted regions were removed and replicates were averaged using the deeptools 66 bigwigCompare –operation mean. Merged and cleaned bigwig files were plotted using the deeptools commands computeMatrix, plotProfile, and plotHeatmap. Heatmaps showing pairwise Spearman correlation between various epigenetic marks in Fig. 5 and Supplementary Fig. S4B were generated with deeptools 66 using replicate merged BAM files. To generate compartment-wise heatmaps, bed files of previously identified compartments at 500 kb resolution were divided in 5 kb bins using the bedtools 63 command makewindows and supplied to deeptools command multiBamSummary and plotCorrelation with default settings.

### RNA-seq analysis

RNA seq analysis was carried out for published data of WT and KO, MEFs and induced neurons from MEFs, as well as osteoblast differentiated MEFs at days 4 and 14, as described in [2]. DEseq was used to perform differential expression analysis between WT/KO of the different studies. Raw counts for WT and β-actin KO cells were downloaded from GSE95830, and DESeq2 56 was used to perform pairwise differential expression comparisons between WT/KO cells. Log2 fold change for genes overlapping different ATAC-Seq clusters was averaged. For repeat element analysis, repeat element annotation for the mouse genome was downloaded from the repeatmasker website (www.repeatmasker.org/genomes/mm10/RepeatMasker-rm405-db20140131/mm10.fa.out.gz) and filtered to exclude simple and low complexity repeats. RNA-Seq data were aligned to the genome using bowtie2 and analysis of repeat elements was performed using the Repenrich2 57 pipeline using default settings followed by a differential analysis of the resulting fraction_counts.txt files using edgeR 58.

### Differential transcriptomic analysis

Differential transcriptomic analysis was performed using JMP Genomics 10. A Benjamini–Hochberg false discovery rate threshold of 0.1 was used to infer statistical significance.

### ChIRP-seq analysis

Adapter trimming was performed on raw fastq files using Trimmomatic. Surviving reads were aligned against the relevant reference genome (GRCm38) using Burrows-Wheeler Aligner BWA-MEM. Resulting BAM alignments were cleaned, sorted, and deduplicated (PCR and optical duplicates) with PICARD tools. Four bam files for each condition (two replicates each for even and odd probes) were merged into a single file. Bigwig files were generated using deeptools command “bamCoverage -bs 10 -e –ignoreDuplicates –normalizeUsing RPKM.” Peaks were called on the merged files using macs2 with the following parameters: “macs2 callpeak -t merged.bam -q 0.05 -g mm -f BAMPE -c input.bam –keep-dup all.” Peaks were classified as being unique to WT, KO or shared with the HOMER command “mergePeaks” and annotated with HOMER script “AnnotatePeaks.pl.” Peaks overlapping blacklisted regions were removed using bedtools. Subsequent analysis was performed using custom R scripts. ChIRP-seq data have been deposited in the Gene Expression Omnibus with accession number GSE262113. Annotated ChIRP-seq peaks are found in (Supplementary Table S5).

### Integrative transcriptomic-CHIRP-seq pathway enrichment analyses

In total, 1551 genes were identified to be bound to *MEG3* in actin (KO) replicates compared to WT replicates. Subsequently, we used the differential gene expression data (fold change and adjusted *P-*value) for each of these genes between the KO and WT conditions generated by transcriptomic profiling to perform a metabolic pathway overrepresentation analysis using Ingenuity Pathway Analysis (IPA; Qiagen).

### GO term analysis

All GO term analyses were performed, and figures generated using ShinyGO.

### Large-scale ultra-performance liquid chromatography high-resolution mass spectrometry

Five replicates of Actin WT, HET, and KO cells were used for metabolomic profiling. Approximately 3 × 10^5^ cells per sample were washed twice with ice-cold 0.9% NaCl solution and 300 μl of 100% methanol was added to each well. After 3 min of incubation on ice, cells were scraped using a precooled cell scraper and moved to a cold Eppendorf tube. Cell extracts were centrifuged at 15 000 rpm for 15 min at 4°C, and 200 μl of each supernatant was moved to a new tube for further processing. Next, samples were vacuum dried and reconstituted in 200 μl of cyclohexane/water (1:1) solution for analysis by Ultra Performance Liquid Chromatography High-Resolution Mass Spectrometry (UPLC-HRMS) performed at the VIB Metabolomics core facility (Belgium). Ten microliters of each sample were injected on a Waters Acquity UHPLC device connected to a Vion HDMS Q-TOF mass spectrometer. Chromatographic separation was carried out on an ACQUITY UPLC BEH C18 (50 × 2.1 mm, 1.7 μm) column from Watersunder under the constant temperature of 40°C. A gradient of two buffers was used for separation: buffer A (99:1:0.1, water:acetonitrile:formic acid, pH 3) and buffer B (99:1:0.1, acetonitrile:water: formic acid, pH 3), as follows: 99% A for 0.1 min decreased to 50% A in 5 min, decreased to 30% from 5 to 7 min, and decreased to 0% from 7 to 10 min. The flow rate was set to 0. 5 ml min^− 1^. Both positive and negative electrospray ionization (ESI) were applied to screen for a broad array of chemical classes of metabolites present in the samples. The LockSpray ion source was operated in positive/negative ESI mode under the following specific conditions: capillary voltage, 2.5 kV; reference capillary voltage, 2.5 kV; source temperature, 120°C; desolvation gas temperature, 600°C; desolvation gas flow, 1000 l h^−1^; and cone gas flow, 50 l h ^− 1^. The collision energy for the full MS scan was set at 6 eV for low energy settings, for high energy settings (HDMSe), it was ramped from 28 to 70 eV. The mass range was set from 50 to 1000 Da, and the scan time was set at 0.1s. Nitrogen (>99.5%) was employed as desolvation and cone gas. Leucine-enkephalin [250 pg/μl solubilized in water:acetonitrile 1:1 (v/v), with 0.1% formic acid] was used for the lock mass calibration, with scanning every 1 min at a scan time of 0.1 s. Profile data were recorded through Unifi Workstation v2.0 (Waters). Data normalization was performed to remove potential variation resulting from instrument inter-run tuning differences. Raw MS peak data representing the abundance of each detected compound were subject to median standardization and missing values were imputed by the minimum value. Compounds with missing values in >50% of samples were considered missing. Standardized data that passed the quality control step were then log2 transformed and IQR normalized using JMP Genomics v8 (SAS Institute, Cary, NC) to remove potential technical artifacts and outliers.

### Metabolomic profiling

Global metabolomic profiling of three replicates from each of the three cell lines was performed using ultra performance liquid chromatography high resolution mass spectrometry (UPLC-HRMS) in positive and negative ionization modes. Overnight grown cells (∼3 × 10^5^ per sample) were washed twice with ice-cold 0.9% NaCl solution and 300 μl of 100% methanol was added to each well. After 3-min incubation on ice, cells were scraped using precooled cell scraper and moved to cold Eppendorf tube. Cell extracts were spun down at 15 000 rpm for 15 min at 4°C and 200 μl of each supernatant moved to a new tube for further processing. Next, samples were vacuum dried and reconstituted in 200 μl of cyclohexane/water (1:1) solution for subsequent analysis by UPLC-HRMS performed at VIB Metabolomics core facility (Belgium). Ten microliters of each sample were injected on a Waters Acquity UHPLC device connected to a Vion HDMS Q-TOF mass spectrometer. Chromatographic separation was carried out on an ACQUITY UPLC BEH C18 (50 × 2.1 mm, 1.7 μm) column from Watersunder under the constant temperature of 40°C. A gradient of two buffers was used for separation: buffer A (99:1:0.1, water:acetonitrile:formic acid, pH 3) and buffer B (99:1:0.1, acetonitrile:water:formic acid, pH 3), as follows: 99% A for 0.1 min decreased to 50% A in 5 min, decreased to 30% from 5 to 7 min, and decreased to 0% from 7 to 10 min. The flow rate was set to 0. 5 ml min^−1^. Both positive and negative ESI were applied to screen for a broad array of chemical classes of metabolites present in the samples. The LockSpray ion source was operated in positive/negative ESI mode under the following specific conditions: capillary voltage, 2.5 kV; reference capillary voltage, 2.5 kV; source temperature, 120°C; desolvation gas temperature, 600°C; desolvation gas flow, 1000 l h^−1^; and cone gas flow, 50 l h^−1^. The collision energy for full MS scan was set at 6 eV for low energy settings; for high energy settings (HDMSe), it was ramped from 28 to 70 eV. Mass range was set from 50 to 1000 Da, and the scan time was set at 0.1s. Nitrogen (>99.5%) was employed as desolvation and cone gas. Leucine-enkephalin [250 pg/μl solubilized in water:acetonitrile 1:1 (v/v), with 0.1% formic acid] was used for the lock mass calibration, with scanning every 1 min at a scan time of 0.1 s. Profile data were recorded through Unifi Workstation v2.0 (Waters). Metabolomic data are publicly available in MendeleyData.

### Statistical analysis of the metabolomic data

Data normalization was performed to remove potential variation resulting from instrument inter-run tuning differences. Raw MS peak data representing the abundance of each detected compound were subject to median standardization and missing values were imputed by the minimum value. Compounds with missing values in >50% of samples were considered missing. Standardized data that passed quality control step were then log2 transformed and IQR normalized. Principal component analysis (PCA) and hierarchical clustering were done to explore the correlation structure in the data across the three conditions (WT, HT, and KO). Furthermore, metabolite-by-metabolite analysis of covariance was performed to investigate differences between actin KO and WT conditions. A Benjamini–Hochberg false discovery rate threshold of 0.1 was used to infer statistical significance. Supervised and unsupervised data analyses were performed using JMP Genomics v10 (SAS Institute, Cary, NC).

### Functional and metabolic pathway enrichment analysis

Functional analysis of curated normalized peak data was performed using MetaboAnalyst v5.0 using an existing protocol [38]. Implemented Gene Set Enrichment Analysis (GSEA) method in the Functional Analysis module of MetaboAnalyst v5.0 (accessed in 2022 from http://www.metaboanalyst.ca/) was used to identify sets of functionally related compounds and evaluate their enrichment of potential functions defined by metabolic pathways. *m/z* values and retention time dimensions both were used to identify and annotate compounds, and improve the accuracy of functional interpretations. Annotated compounds were then mapped onto Mus musculus (mouse) [KEGG] [39], for pathway activity prediction (Supplementary Table S3). GSEA calculates Enrichment score (ES) by walking down a ranked list of metabolites, increasing a running-sum statistic when a metabolite is in the metabolite set and decreasing it when it is not. A metabolite set is defined in this context as a group of metabolites with collective biological functions or common behaviors, regulations or structures. In this method, a metabolite set are defined in this context as a group of metabolites with collective biological functions or common behaviors, regulations or structures. Annotated metabolites were then mapped onto Mus musculus (mouse) (KEGG) (https://pubmed.ncbi.nlm.nih.gov/12466850/) for pathway activity prediction.

### Formaldehyde crosslinking-RNA immunoprecipitation

Actin WT and KO MEFs were harvested by centrifugation at 500 RCF for 5 min, cells were rinsed with 1× PBS at room temperature. Each 5 million cells were resuspended in 1 ml of FBS and high salt free media. Subsequently cells were crosslinked for 10 min with 0.1% methanol free formaldehyde (Sigma) as per Hendrickson *et al.* [40, 41]. Quenched with 125 mM of glycine. Cells were centrifuged again at 500 RCF for 5 min followed by two washes with cold PBS. The resulting pellets were flash-frozen in liquid nitrogen and stored at −80°C for future use.

Upon thawing, the frozen pellets were resuspended in 1 ml of RIPA lysis buffer per tube (consisting of 50 mM Tris pH 8.0, 150 mM KCl, 0.1% SDS, 1% Triton X-100, 5 mM EDTA, and 0.5% sodium deoxycholate), supplemented with freshly prepared 0.5 mM dithiothreitol (DTT), 1× EDTA protease inhibitor cocktail (Roche), and 100 U/ml RNaseOUT (Life Technologies, 10777-019). The cells were then incubated while rotating at 4°C for 10 min, followed by sonication by the Qsonica Sonicator at 10% amplitude for 1 s on and 1 s off at 30-s intervals, for a total of 90 s. The lysate was centrifuged at maximum speed for 10 min at 4°C, and the supernatant was collected. An equal volume of fRIP binding/wash buffer (150 mM KCl, 25 mM Tris pH 7.5, 5 mM EDTA, and 0.5% NP-40), supplemented with freshly prepared 0.5 mM DTT, 1× EDTA protease inhibitor cocktail (Roche), and 100 U/ml RNaseOUT (Life Technologies, 10777-019), was added to dilute the samples. Fifty microliters of the lysate were set aside as the input sample and stored at −20°C for subsequent RNA purification and library preparation.

For pre-clearing, each lysate of 5 million cells was incubated with 25 μl of Pierce Protein A/G Magnetic Beads (Thermo Scientific) on a rotor at 4°C for 30 min. Aliquots of 5 million cells/ml were flash-frozen and stored at −80°C for future use.

To initiate the fRIP procedure, the lysates were thawed, and 5 μg of H3K27ac antibody (ChIP grade- ab4729), H3k27me3 antibody (C36B11 Cell Signaling), and H3K9me3 antibody (ChIP grade ab8898) were added. The lysates, along with the antibody, were left to rotate at 4°C overnight before the addition of Pierce Protein A/G Magnetic Beads (Thermo Scientific). Fifty microliters of beads were added per 1 ml of lysate for 1 h to recover immunoprecipitated complexes. Washes were performed twice using 1 ml of fRIP binding/washing buffer. After the final wash, the beads were removed and stored at −20°C. fRIP was then followed by RNA purification, reverse transcription to cDNA (complementary DNA) (Qiagen quantitect reverse transcription kit- cat# 205 311), and RT-qPCR.

### *Meg3* Knockdown

*Meg3* was knocked down in WT MEFs and β-actin KO MEFs using customized antisense LNA Gapmers, from Qiagen.

### RNA-seq data analysis upon *Meg3* Knockdown

Demultiplexed using BCL2FASTQ version 2.20.0.422 allowing up to one mismatch in the barcode sequence and adapter filtering as the reads are demultiplexed. Raw FASTQ sequenced reads were first assessed for quality using FastQC v0.11.5. The reads were then passed through Trimmomatic v0.36 for quality trimming and adapter sequence removal, with the parameters (ILLUMINACLIP: trimmomatic_adapter.fa:2:30:10 TRAILING:3 LEADING:3 SLIDINGWINDOW:4:15 MINLEN:36). The surviving trimmed read pairs were then processed with Fastp in order to remove poly-G tails and Novaseq/Nextseq specific artefacts. The quality trimmed reads were aligned to the mouse reference genome GRCm38.82 using HISAT2 with the default parameters and additionally by providing the –dta flag. The resulting SAM alignments were then converted to BAM format and coordinate sorted using SAMtools v1.3.1. Raw counts for WT and β-actin KO cells were then merged and processed using NASQAR to generate the PCA and box plots.

### Statistical tests

Statistical significance was calculated using GraphPad Prism for macOS, GraphPad Software, La Jolla California USA, www.graphpad.com.

### Data availability

ChIRP-seq data have been deposited in the Gene Expression Omnibus with accession number GSE262113 (token alypaokcvhuhfcf). For Ezh2 ChIP-seq analysis bigwig was downloaded from Gene Expression Omnibus with accession number GSE149987 (public). For BRG1, H3K9me3, and H3K27me3 ChIP-seq analysis, bigwig files were downloaded from GEO accession number GSE100096 (public). RNA-seq datasets upon *Meg3* knockdown are publicly available in the SRA database under the accession number PRJNA1131122 (public). The mass spectrometry proteomics data have been deposited to the ProteomeXchange Consortium via the PRIDE (59) partner repository with the dataset identifier PXD051730 and 10.6019/PXD051730 (for reviewers, Username: reviewer_pxd051730@ebi.ac.uk; Password: W7wtLeZj). The link to a UCSC genome browser session displaying the uploaded sequence tracks can be found at UCSC genome link.

## Results

### Nuclear β-actin depletion leads to increased expression of the lncRNA *Meg3*

**Figure 1.**
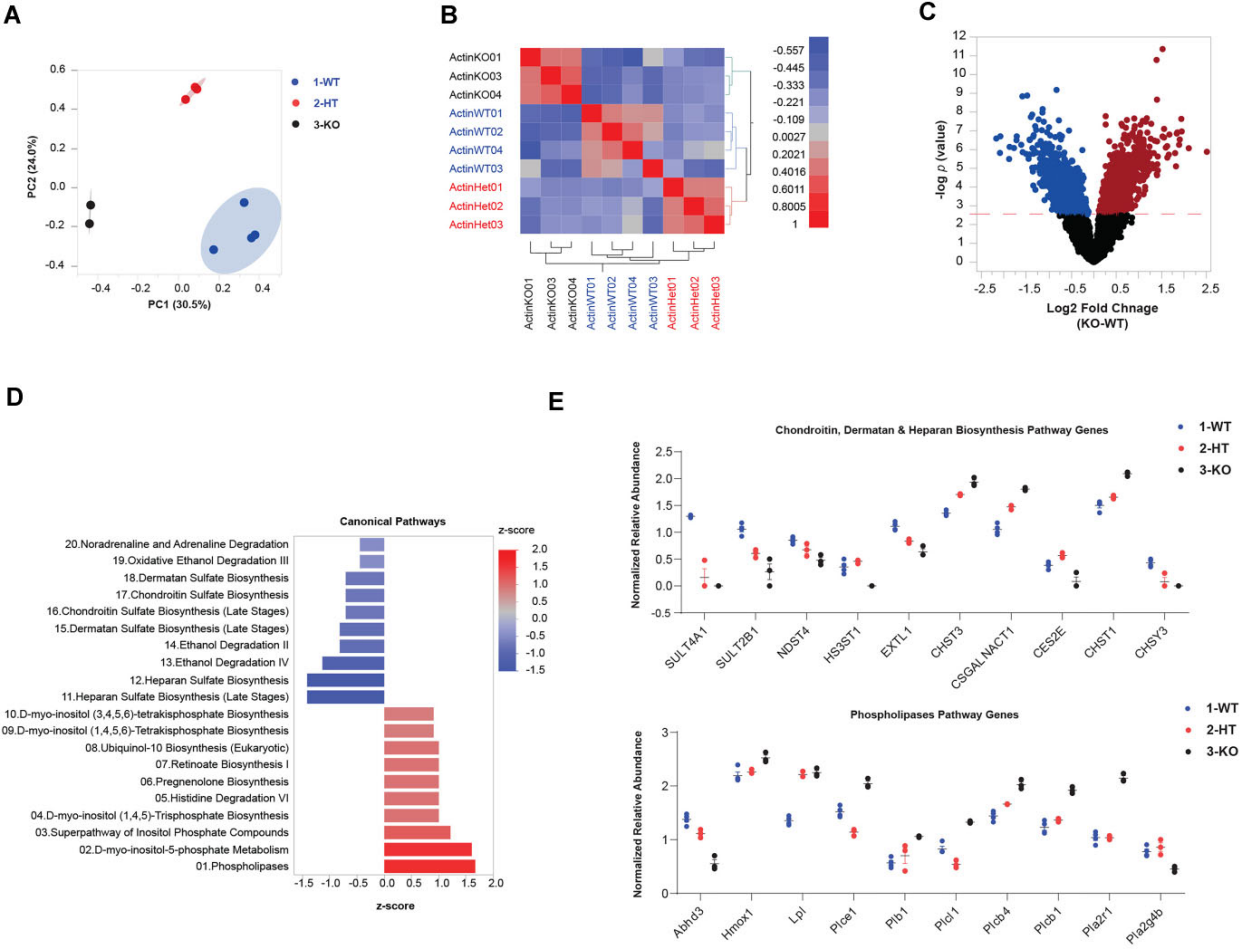
**Transcriptional profiling by RNA-seq analysis shows significant differential gene expression upon β-actin depletion**. (**A**) PCA showing the differential correlation of WT, HET and β-actin KO conditions; separation is highly noted between WT, HET, and KO. Prin1 represents the highest common variable 1 (30.5%) and Prin2: the second most common variable (24.0%). The PCA plot visualizes the relationship and variability among the WT, HET, and β-actin KO samples based on their genomic RNA-seq data. Each replicate for each sample is represented by a data point in the plot, and the position of the data points reflects their similarity or dissimilarity in gene expression profiles. (**B**) The hierarchical clustering heatmap represents the correlation analysis of genomic RNA-seq data from three samples: WT, HET, and β-actin KO and their replicates. Each row and column in the heatmap correspond to a specific sample replicate. The different intensity represents the correlation coefficient between the expression profiles of two samples, lowest = −0.5 and highest = 1. (**C**) The volcano plot showing the differential expression between the transcriptomic profiles of KO and WT, significantly upregulated and downregulated genes are highlighted. (**D**) Pathway enrichment analysis showing the most significantly enriched metabolic pathways in the whole RNA seq datasets. the pathways are ranked by the fold change. Upregulated and downregulated pathways are shown by different Z scores and shade intensities. (**E**) Two one-way plots that show the differential expression of genes implicated in two pathways (upper panel; chondroitin, heparan, and dermatan biosynthesis pathways) and (lower panel; phospholipases genes). Interestingly, most of the genes implicated in these pathways show a dosage dependent effect. Wild-type (WT), Heterozygous (HT), and Knock out (KO).

To find out if β-actin depletion affects expression of lncRNAs, we analyzed RNAseq data sets obtained in fibroblasts from WT, HET, and β-actin KO mouse embryos, hereby referred to as WT MEFs, HET MEFs, and KO MEFs [9, 10, 23]. As expected, results from PCA on the normalized datasets show clear segregation among replicates of WT, KO, and HET MEFs as revealed by first (PC1) and second principal components (PC2), explaining 54.5% of the variation among the samples (Fig. 1A). Clustering of the transcriptomic correlation matrix based on similarity confirmed that replicates of each condition cluster together and β-actin deletion leads to changes in gene expression (Fig. 1B). To identify gene expression alterations associated with β-actin deletion, we performed gene-by-gene analysis of covariance focusing on the comparison between β-actin KO and WT, which included 46 603 genes. The analysis revealed 718 differentially expressed genes (FC ≥ |1.5|, B-H FDR < 0.05) (Fig. 1C), 359 (50%) of which are downregulated in the β-actin KO condition.

Next, we investigated the functional characteristics of the genes with significantly altered expression levels in the β-actin KO condition using IPA. Remarkably, we found significant downregulation of metabolic pathways and six of the most downregulated ones are involved in chondroitin sulfate, heparan sulfate, and dermatan sulfate biosynthesis (heparan sulfate biosynthesis late stages, heparan sulfate biosynthesis, dermatan sulfate biosynthesis late stages, dermatan sulfate biosynthesis, chondroitin sulfate biosynthesis late stages, and chondroitin sulfate biosynthesis) (Fig. 1D). Out of ten genes involved in the three aforementioned pathways, eight follow a significant β-actin dosage-dependent expression. Those genes upregulated in the KO condition include CSGALNACT1, CHST3, and CHST1, while SULT4A1, SULT2B1, NDST4, EXTL1, CES2E, and CHSY3 are downregulated. Also, ethanol degradation pathways II, III, and IV were found to be significantly downregulated. In contrast, the phospholipases pathway represents the most significantly upregulated one. Seven out of the 10 genes involved in the phospholipases pathway are β-actin dosage dependent. In particular, Abhd3 and pla2g4b are downregulated whereas Hmox1, Lpl, Plb1, Plcb4, and Plcb1 are upregulated (Fig. 1E). This highlights the impact of β-actin deletion on gene expression in these pathways. Notably, the transcriptomic changes that exhibited a dosage-dependent effect, with progressive alterations observed from WT to HET and KO MEFs confirm what was previously identified [13] and suggest a potential disruption of key metabolic pathways.

**Figure 2.**
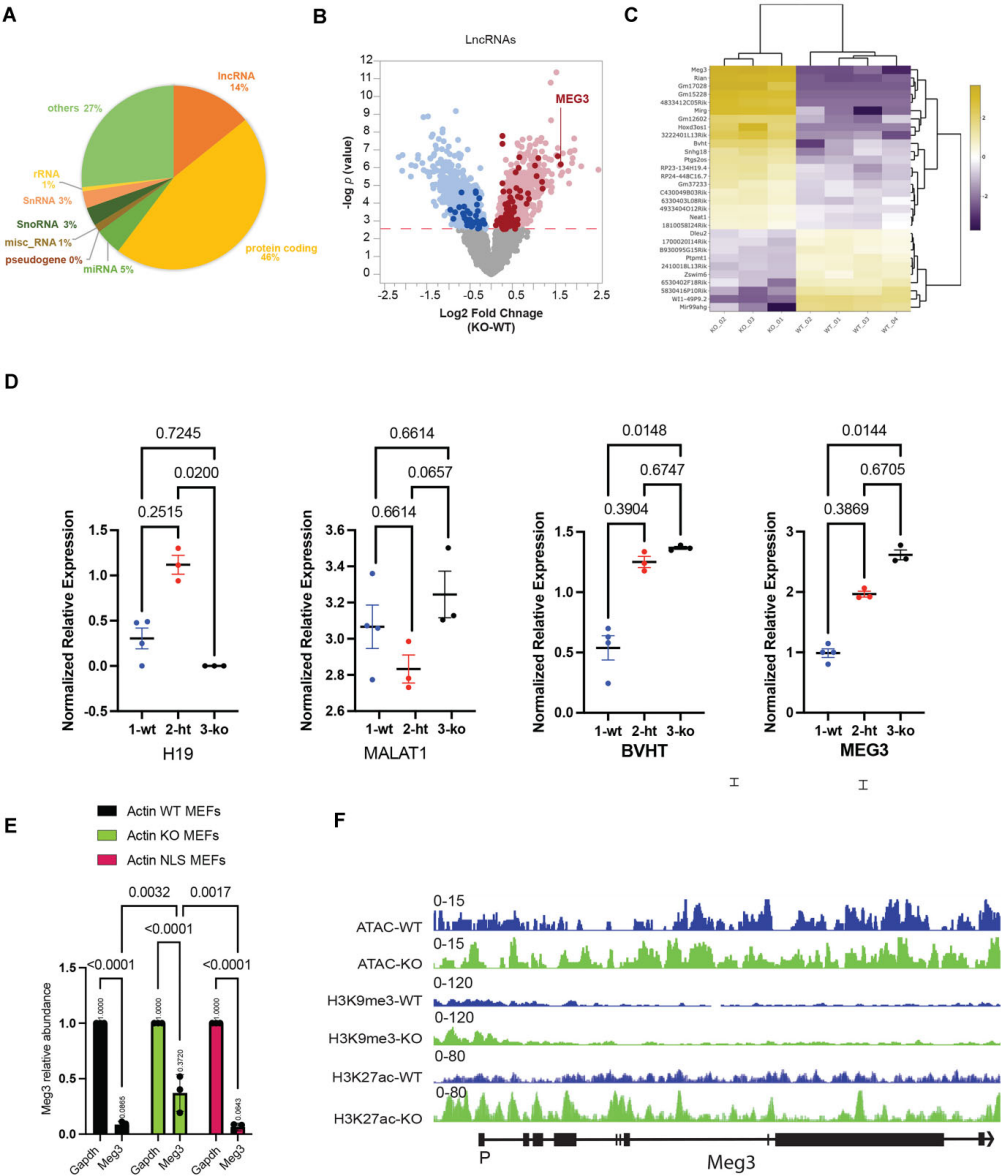
***Meg3* lncRNA expression is significantly upregulated upon nuclear β-actin depletion**. (**A**) A pie chart showing the percentages of different RNA types differentially dysregulated upon β-actin KO where protein coding RNAs are 46%, lncRNAs are 14%, miRNAs 5%, SnoRNA 3%, SnRNA 3%, rRNA and miscRNA are 1% each, and others are 27%. (**B**) Volcano plot showing differential expression, of all genes of all lncRNAs only in WT and KO MEFs. *P*-values based on two-tailed Wald test corrected for multiple testing using Benjamini–Hochberg procedure. The significantly upregulated and downregulated lncRNAs are highlighted. Also, the most upregulated lncRNA, *Meg3* is labeled. (**C**) Heatmap of the top 30 differentially expressed candidate lncRNA. (**D**) One-way boxplots showing the differential expression of *Meg3*, *Malat1*, *Bvht* and *H19* in WT, HET, and KO. (**E**) Rt-qPCR showing the *Meg3* relative abundance versus *Gapdh* in WT, KO, and in NLS. Data are shown as mean ± SD (*n* = 3). *P-*values indicated are based on two-way ANOVA multiple comparisons in GraphPad Prism version 10. (**F**) IGV of *Meg3* gene, showing ATAC-seq, H3K9me3 and H3K27ac ChIP-seq data in WT and β-actin KO conditions. The reference gene body for Meg3 is shown at the bottom.

In metabolic diseases, expression of lncRNAs is dysregulated [42] and in cancer, lncRNAs act can act oncogenes and tumor suppressors. They perform some of these functions bound to polycomb repressive complexes or through interactions with transcription factors and DNA methyltransferases [43, 44]. We, therefore, hypothesized that lncRNAs expression and function are potentially dependent on nuclear β-actin. Results from RNAseq analysis confirmed that there is a total of 6622 differentially expressed lncRNAs, accounting for 14% of the total 46,603 generated RNAs (Fig. 2A). Other noncoding RNAs, such as miRNAs, SnRNAs, SnoRNAs, and rRNAs, each comprised <5% of the total set of sequenced RNAs (Fig. 2A). Among the differentially expressed lncRNAs, 36 were downregulated and 85 were upregulated (Fig. 2B). Since their expression levels are known to increase upon metabolic and cellular stress, we next focused on the set of upregulated lncRNAs in KO cells in relation to WT condition. The ANCOVA analysis revealed *Meg3* as the most upregulated lncRNA (fold change = 3.09, *P*= 0.0002) (Fig. 2B and C) and upregulation appears to be β-actin dosage dependent with KO MEFs having the highest expression compared to HET and WT (Fig. 2D). We also examined the expression of well-studied candidate lncRNA genes involved in transcription regulation and/or chromatin organization, such as Bvht, Malat1, and H19. Bvht exhibited increased expression in the KO compared to WT but to a lesser extent than *Meg3*, while Malat1, known for its involvement in speckle formation and gene silencing through interactions with the Ezh2 and Eed Polycomb subunits, showed no significant change [41, 45, 46]. In contrast, H19 displayed a significant decrease in the KO compared to WT (Fig. 2D). Notably, the significant upregulation of *Meg3* upon β-actin depletion was rescued by reintroduction of an NLS-tagged β-actin in the cell nucleus of β-actin KO MEFs (Fig. 2E). *Meg3* changes in gene expression are likely to occur due to changes in the chromatin landscape across the gene locus. ATAC-seq and ChIPseq analyses showed that in the absence of β-actin there is an increase in chromatin accessibility across the *Meg3* gene and promoter region accompanied by a general increase in H3K27 acetylation levels (Fig. 2F).

Together, these results suggest that *Meg3* expression is directly regulated by the nuclear β-actin pool through a chromatin-based mechanism that regulates H3K27 acetylation levels.

### Actin dependent *Meg3* enrichment at promoters/TSS of metabolic genes contributes to metabolic alterations

**Figure 3.**
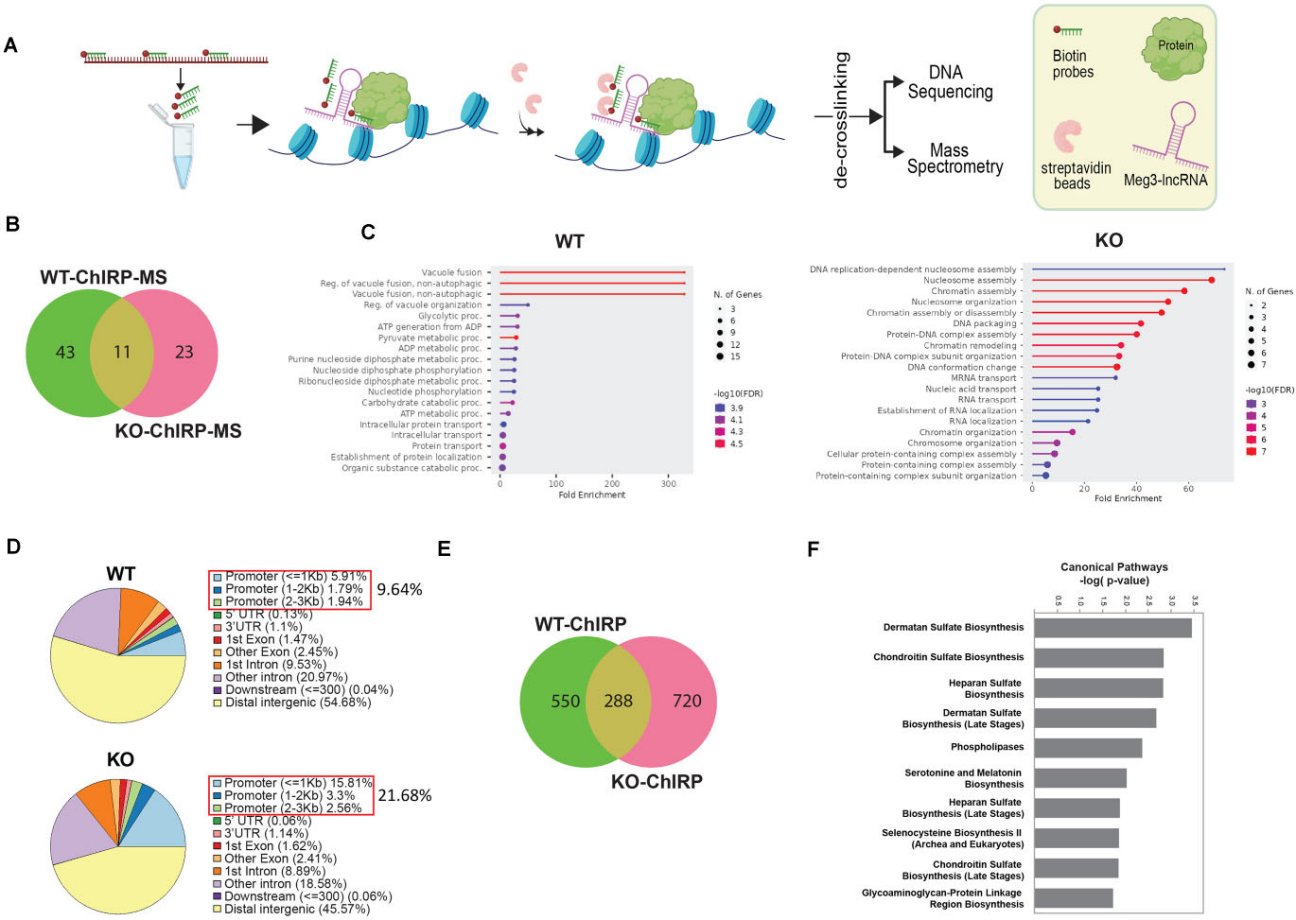
**Genome-wide association of *Meg3* lncRNA is altered in the absence of β-actin**. (**A**) The ChIRP protocol followed to pull down *Meg3* lncRNA using biotinylated probes and streptavidin beads (Created in BioRender. Percipalle, P. (2025) https://BioRender.com/qmhj5mh, adapted from [47]). (**B**) Venn diagram showing number of WT ChIRP-MS protein hits and the KO-ChIRP-MS hits. (**C**) GO Analysis showing the biological processes of proteins bound to *Meg3* in actin WT MEFs and actin KO MEFs. (**D**) Pie plots to visualize the genomic annotation of Peaks in WT and KO showing percentages of the annotated peaks of *Meg3* loci on the genome. (**E**) Venn diagram showing the 550 specific peaks of *Meg3* bound genes in WT (WT-ChIRP) and 720 specific peaks of *Meg3* bound genes in KO (KO-ChIRP), while common peaks between *Meg3*-WT-ChIRP and *Meg3*-KO-ChIRP are equal to 288. (**F**) An overrepresentation pathway analysis using the list of genes that have been shown to bind to *Meg3* in KO relative to those in WT. Remarkably, the results show significant enrichment of chondroitin, heparan, and dermatan biosynthesis pathways as well phospholipases. Statistical significance is determined using right-tailed Fisher’s exact *t*-test and B–H FDR < 0.1. The significance of enrichment analysis represented using −log (*P-*value) is plotted on the *x-*axis.

We next studied whether *Meg3* associates with the chromatin, across the genome, and whether this potential association changes upon β-actin depletion using a combination of chromatin isolation by RNA Purification followed by mass spectrometry (ChIRP-MS) and chromatin isolation by RNA purification followed by deep sequencing (ChIRP-seq) [47] (Fig. 3A). For this, we designed *Meg3* specific biotinylated probes and used them to pull down chromatin-bound *Meg3* from crosslinked MEFs using streptavidin beads (Fig. 3A). After mass spectrometry analysis, we identified a set of 54 proteins associated with *Meg3* in the WT condition and 34 in the KO condition. Eleven protein targets were common to both WT and KO condition, 43 were specific to WT and 23 were specific to KO (Fig. 3B). Gene ontology (GO) of the WT revealed that proteins associated with chromatin-bound *Meg3* are involved in energy production and metabolic processes such as vacuole fusion, glycolytic processes, ATP generation from ADP, and pyruvate metabolic processes among many other metabolic terms at an enrichment of up to 300-fold (Fig. 3C). On the other hand, in the β-actin KO condition, results from GO term analysis revealed that *Meg3* primarily binds to proteins involved in DNA replication-dependent nucleosome assembly, nucleosome assembly, chromatin assembly, nucleosome organization and other chromatin organization, and assembly-related biological processes with an enrichment of over 70-folds (Fig. 3C). Compatible with this analysis, in the KO condition, the ChIRP MS revealed loss of actin association and significant high confidence enrichment of all histones, including H2B, H3, and H4, being present in the fraction of proteins associated with chromatin-bound *Meg3* (Supplementary Table S1). These results confirm that *Meg3* associates with the chromatin and that this interaction is likely to be enhanced in the β-actin KO condition when results from ChIRP-MS experiments show *Meg3* directly interacts with histones. ChIRP combined with deep sequencing (ChIRP-seq) further confirmed this hypothesis, identifying a broad association of *Meg3* with the WT mouse genome that is significantly altered in β-actin KO across all chromosomes (Supplementary Fig. S1). We found an overall number of 720 peaks in the KO condition in comparison to 550 in the WT condition and 288 peaks common to both (Fig. 3E) with a slight increase of *Meg3* association with promoters/TSS in β-actin KO MEFs (Supplementary Fig. S2A and B). Although the majority of *Meg3* preferentially binds distal intergenic regions in both WT and KO cells (Supplementary Fig. S3A and B), significant *Meg3* binding at promoters was also observed in the KO cells (Supplementary Fig. S4). In WT MEFs *Meg3* comprise ∼9.64% at promoters, the majority of which are promoters that are ≤1 kb (5.91%). In the KO condition, 21.68% of ChIRP-seq peaks were found at promoters, mostly promoters ≤1 kb (15.82%) (Fig. 3D). Deeper analysis using upset plots showed the intersection of peaks among the different regions of the genome, where among the many intersections between genic, intergenic and distal intergenic regions, there is a noticeable increase in the promoter intersections from 9 to 13 intersections of peaks upon β-actin KO (Supplementary Fig. S2D and E). Metabolic pathway overrepresentation analysis by IPA for 1551 genes were identified to be bound to *Meg3* in β-actin KO replicates compared to the WT replicates. Chondroitin, heparan, and dermatan biosynthesis and phospholipases pathways were the most significantly overrepresented pathways (Fig. 3F). These results suggest that in β-actin KO cells *Meg3* increased binding to metabolic genes may affect gene activity and lead to alterations in metabolic pathways.

**Figure 4.**
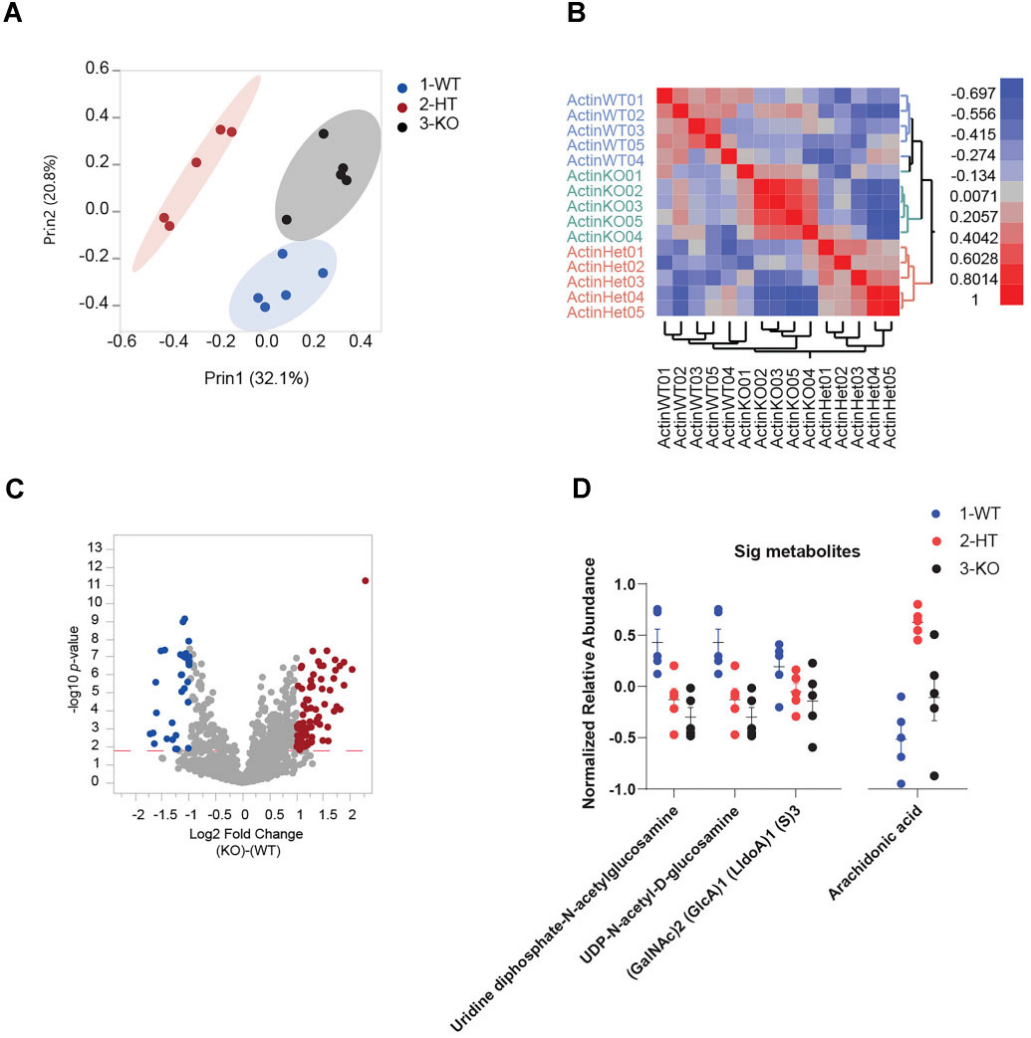
β-Actin dosage dependent dysregulation of key metabolic pathways and metabolites. (**A**) PCA demonstrating that WT, HT, and KO samples show distinctive clustered metabolic profiles. (**B**) Hierarchical clustering analyses demonstrating that WT, HT, and KO samples show distinct metabolic profiles. (**C**) A volcano plot demonstrating the differential expression of the normalized abundance of all the metabolic features between KO and WT. Significantly upregulated and downregulated metabolic features are highlighted. (**D**) A one-way plot for three different metabolites implicated in the chondroitin, heparan, and dermatan pathway being downregulated in HT and KO compared to WT. It also shows another one-way plot for arachidonic acid, one of the main byproducts of phospholipase activity, being unregulated in HT and KO compared to WT.

To further investigate perturbations in chondroitin, heparan, and dermatan biosynthesis and phospholipases pathways in association with the loss of actin and, potentially, *Meg3* upregulation, we performed a global metabolomic profiling of the three cell lines in three replicates using positive and negative ionization (see Materials and methods section). A total of 2499 metabolic features were detected in all samples and replicates (*n* = 3). *m/z* values and retention time were recorded for each metabolic feature and used for further analysis (see Materials and methods section). PCA of the normalized peak dataset revealed a strong correlation structure in the metabolomic data across both conditions with the first principal component (PC1) clearly capturing the effect of partial and complete loss of function in actin and showing clear segregation between replicates of the WT, HET, and KO conditions (Fig. 4A). The first two principal components explain 52.9% of the variation in the dataset (Fig. 4A). Similarly, two-way hierarchical clustering of the full metabolomic dataset including all replicates and all metabolic features (*n* = 2499) displays discrete metabolic footprints for both the actin KO and actin HET from that of the WT condition (Fig. 4B).

Next, we performed metabolite-by-metabolite analysis of covariance focusing on the comparison between actin KO and WT. The analysis revealed 501 significantly differentially abundant features in actin KO compared to the WT (FC ≥ 1.5, B–H FDR < 0.1, Fig. 4C). A summary of the metabolic features with statistically significant differential abundance in actin KO is shown in Supplementary Table S2. Together, these results clearly show the strong effect of β-actin loss on the cell metabolome and hint to major perturbations in metabolic resources allocation in the absence of β-actin. Next, we performed a functional analysis of curated normalized peak data using GSEA method implemented in MetaboAnalyst v5.0 to identify and annotate sets of functionally related compounds and evaluate their enrichment of potential functions defined by metabolic pathways. GSEA analysis of compounds identified using positive and negative ionization was performed separately. Putative annotation of MS peaks data was performed using *m/z* values and retention time dimensions to increase confidence in compounds identities and improve the accuracy of functional interpretations. In this method, a metabolite set is defined in this context as a group of metabolites with collective biological functions or common behaviors, regulations or structures. Annotated compounds were then mapped onto Mus musculus (mouse) (KEGG) (https://pubmed.ncbi.nlm.nih.gov/12466850/) for pathway activity prediction.

Of the metabolites implicated in the chondroitin, heparan, and dermatan biosynthesis, we highlight UDP-GlcNAc and (GalNAc)2 (GlcA)1 (LldoA)1 (S)3. UDP-GlcNAc is extensively involved in intracellular signaling as a substrate for *O*-linked *N*-acetylglucosamine transferases (OGTs) to install the O-GlcNAc post-translational modification in a wide range of species. It is also involved in nuclear pore formation and nuclear signalling. OGTs and OG-ases play an important role in the structure of the cytoskeleton. Also, (GalNAc)2 (GlcA)1 (LldoA)1 (S)3 is an amino pentasaccharide and a galactosamine oligosaccharide. Interestingly, the levels of both metabolites showed significant dose–response among the WT, actin HET, and actin KO conditions (Fig. 4D). We also highlight arachidonic acid, one of the main intermediate metabolites of the phospholipases’ pathway and an essential fatty acid that is released from cell membrane by the activity of phospholipase A2 enzymes. Our analysis shows that the levels of arachidonic acids are significantly higher in actin HET and actin KO than in WT (Fig. 4D).

Taken together, the results of transcriptomic, ChIRP-seq and metabolomic data analyses support that the downregulation and upregulation observed in the chondroitin, heparan, and dermatan biosynthesis and phospholipases pathways, respectively, are mediated by the upregulation of *Meg3* upon the loss of β-actin.

### *Meg3* regulates transcription of metabolic genes by affecting promoter–enhancer interactions in a β-actin dependent manner

**Figure 5.**
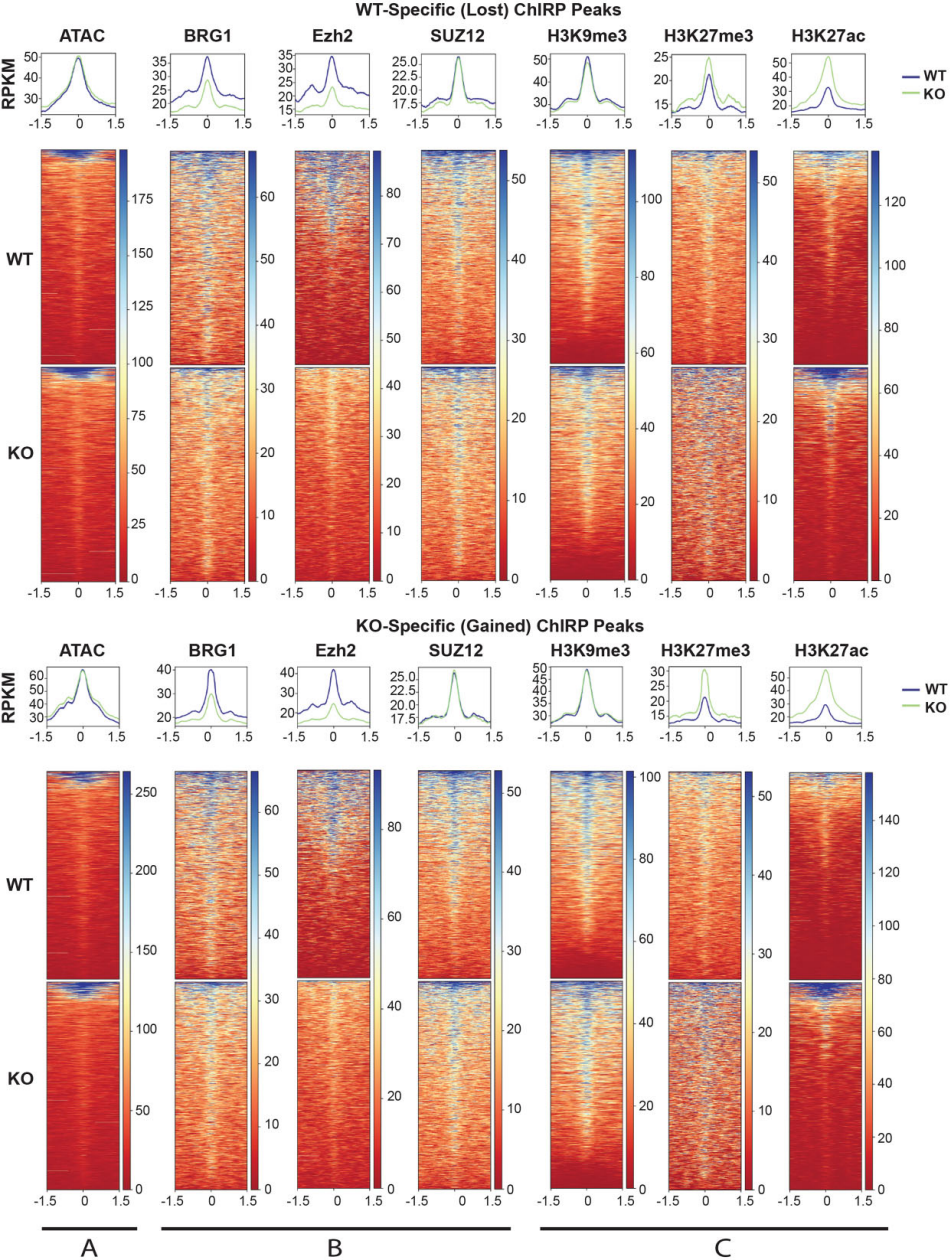
Integration of ChIP-seq, ChIRP-seq, and ATAC-seq data sets shows increase of both active and repressive histone marks in KO-specific *Meg3* ChIRP-seq peaks. Density plots and heatmaps displaying scaled-read densities for ATAC-Seq, *Brg1*, *Ezh2*, *Suz12*, H3K9me3, H3K27me3, and H3K27ac in 3 kb regions surrounding WT-specific (top) and KO-specific (bottom) *Meg3* Peaks. Scale bar shows normalized RPKM.

We next investigated if changes in chromatin accessibility and epigenetic landscape accompany loss (Fig. 5, top panel) or gain (Fig. 5, bottom panel) of *Meg3* ChIRP-seq peaks at genomic sites upon β-actin depletion. For this, we used a combination of ATAC-seq and ChIP-seq with antibodies to the BAF ATPase subunit Brg1, to the Ezh2 catalytic subunit of PRC2 which acts as the repressive H3K27 methyltransferase, and to the Suz12 RNA binding subunit of PRC2, known to be present together with Ezh2 at repressed loci [48]. Results from ATAC-seq did not show changes in chromatin accessibility between WT and KO conditions when comparing regions loss or gain of *Meg3* binding (see Fig. 5A, top and bottom panels) and, similarly, results from ChIP-seq experiments did not show major changes in binding of Brg1, Ezh2, and Suz12 (see Fig. 5B, top and bottom panels). We next performed ChIP-seq analysis using antibodies to H3k27me3, H3K9me3, and H3K27ac, histone modifications respectively mediated by PRC2 and BRG1. Remarkably, the accumulation of H3K27me3 and H3K27ac at sites of differential *Meg3* activity potentially points towards a role for *Meg3* in regulation of chromatin remodeling. While we don’t see significant change in BRG1 and Ezh2 occupancy at differential ChIRP peaks, it is important that we always see distinct peak like pattern of BRG1 and Ezh2 in the heatmaps of ChIRP peaks suggesting the *Meg3* usually colocalizes with these factors. Furthermore, we have shown by ChIRP-MS that *Meg3* also associates with histones and factors involved in nucleosome assembly and reorganization (see in Fig. 3C, right panel). These observations reinforce *Meg3* association with BRG1 and Ezh2 and suggest a functional correlation with H3K27me3 and H3K27ac, are marks of poised and active promoters respectively and we show increased *Meg3* binding at promoter regions in the KO condition.

**Figure 6.**
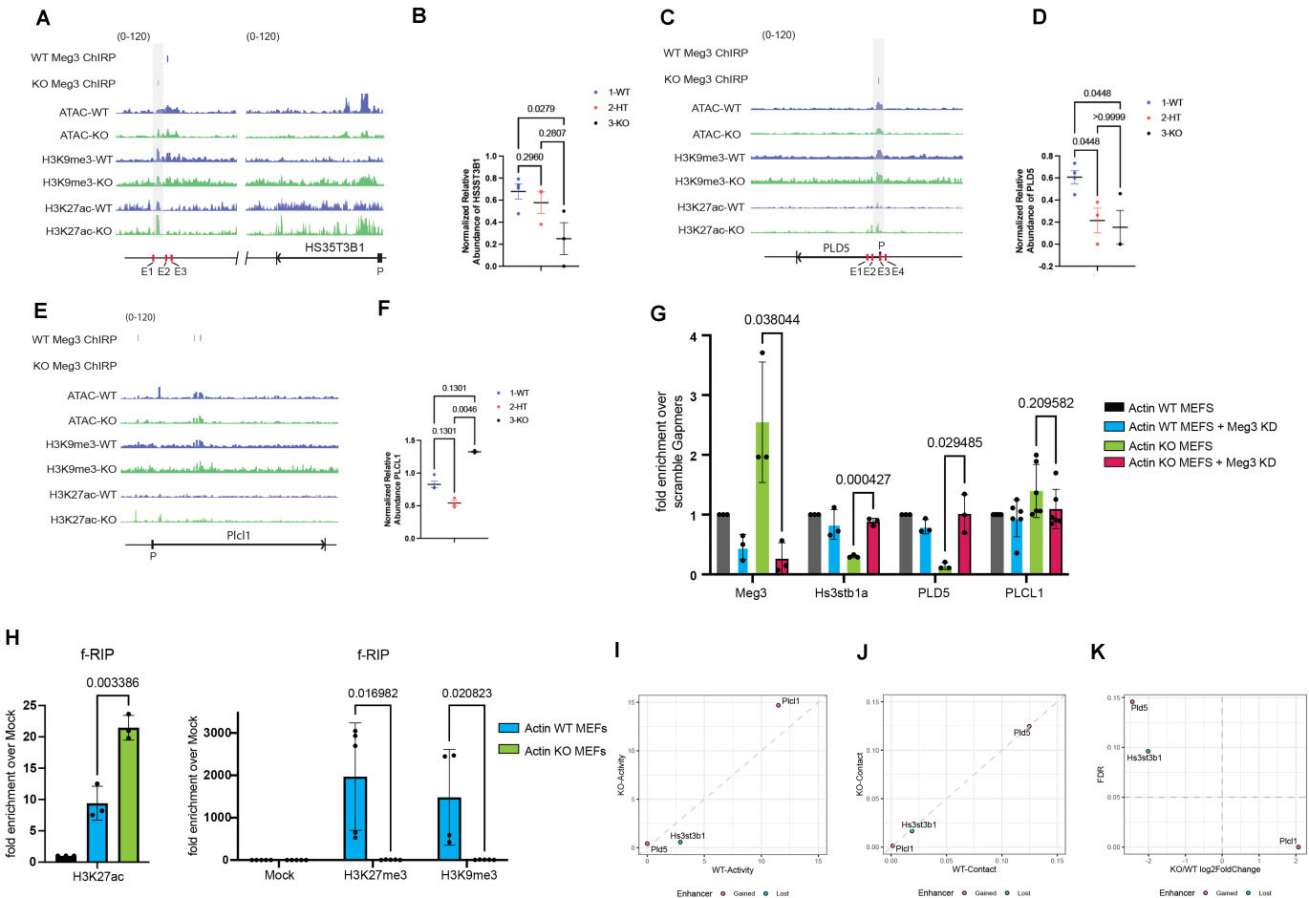
***Meg3* binds to enhancer regions and affects expression of metabolic genes by interfering with promoter–enhancer contacts**. IGVs of *Meg3* ChIRP-seq, ATAC-seq, H3K9me3, and H3K27ac ChIP-seq data in WT and β-actin KO conditions. The reference gene body loci are shown at the bottom for Hs3st3b1 (**A**), Pld5 (**C**), and Plcl1 (**E**) (exon: box, intron: line). The *y*-axis represents RPKM (Reads Per Kilobase of sequence range per Million mapped reads) per bin. The range was set the same as in the image. Plots showing the normalized relative expression of Hs3st3b1 (**B**), Pld5 (**D**), and Plcl1 (**F**) in WT, HT, and KO. (**G**) Rt-qPCR analysis showing the differential enrichment of *Meg3*, Hs3stb1a, PLD5, and Plcl1, in Actin WT MEFs, Actin WT MEFS + *Meg3* KD, Actin KO MEFS, and Actin KO MEFS + *Meg3* KD. Data are shown as mean ± SD (n = 3–6). P-values indicated are based on multiple unpaired t-tests Holm-Šídák method in GraphPad Prism version 10. (H) H3K27 acetylation, H3K27me3, and H3K9me3 f-RIP-qPCR showing *Meg3* enrichment at regions of H3K27ac, H3K27me3, and H3K9me3, respectively in WT and actin KO MEFs. Data are shown as mean ± SD (n = 3–4). P-values indicated are based on multiple unpaired t-tests Holm-Šídák method in GraphPad Prism version 10. Results from the ABC model analysis showing enhancer activity (I), enhancer–promoter contact (J), or fold change (K) of Hs3st3b1, PLD5 or PLCl1 genes.

H3K27ac is a mark of active regulatory regions, including both promoters and enhancers. Its levels are increased upon β-actin depletion. Since in the same condition *Meg3* levels are enhanced at regulatory regions, we next studied if *Meg3* binding to gene regulatory regions functionally correlate with actin-dependent changes in H3K27ac levels. To this end, we focused on those genes that are commonly involved in the chondroitin, heparan, and dermatan biosynthesis and phospholipases pathways and are the most significantly overrepresented pathways in the KO condition. In particular, we focused on the gene encoding HS3ST3B1 (heparan sulfate-glucosamine 3-sulfotransferase 3B1), a type II integral membrane protein that belongs to the 3-*O*-sulfotransferases family, catalyzing the addition of sulfate groups at the 3-OH position of glucosamine in heparan sulfate (Fig. 6A). Remarkably, in the KO condition, we found a new site of *Meg3* enrichment (new ChIRP-seq peak) in intergenic regions, located between two enhancers, referred to as E1 and E2 (Fig. 6A), and this binding seems to correlate with a dosage-dependent repression of the Hs3st3b1 gene (Fig. 6B). Similarly, we found that the *Meg3-*binding site exhibits a specific increase in H3K27ac levels concomitantly with a decrease in the levels of H3K9me3 and marginal changes in ATAC signal (see Fig. 6A and Supplementary Fig. S7).

We also found changes in *Meg3* enrichment peaks in the PLD5 (phospholipase D family member 5) (see Fig. 6C), which is part of the phospholipases biosynthesis pathway [49–51], involved in the conversion of phosphatidyl-choline into 1,2-diacyl-sn-glycerol-3-phosphate (Supplementary Fig. S6). In this case, *Meg3* binds to sites within enhancers very close to the gene promoter, and upon β-actin depletion *Meg3* enrichment correlates with significant increase in H3K27ac levels, no H3K9me3 changes and only marginal ones in ATAC signal (Fig. 6C and Supplementary Fig. S7). Binding of *Meg3* also seems to correlate with dosage dependent decrease in PLD5 mRNA levels (Fig. 6D). In contrast to HS3ST3B1 and PLD5 which are both down-regulated in the absence of β-actin and exhibit association of *Meg3* close to or at regulatory regions such as enhancers and promoters, in the phospholipase C like 1 enzyme gene (PLCL1), involved in converting phosphatidylinositol-4,5-bisphosphate into both the secondary messengers’ diacylglycerol and the inositol-1,4,5-triphosphate (Supplementary Fig. S6), *Meg3* binding does not target regulatory regions (Fig. 6E), it only binds far from enhancers (not shown) which seems to result in PLCL1 over expression in the KO condition (Fig. 6F). To find out if *Meg3* binding directly affects changes in expression of these genes, we next applied *Meg3*-specific gapmers to WT and β-actin KO cells to knock down *Meg3*. qPCR analysis on total RNA isolated from WT and KO cells shows that HS3ST3B1a and PLD5 mRNA levels significantly increase in the absence of *Meg3* in the β-actin KO background, while PLCL1 mRNA levels decrease (Fig. 6G), suggesting that indeed, upon β-actin depletion *Meg3* is directly involved in their transcriptional changes. We next performed RNA sequencing analysis on total RNA isolated from β-actin KO MEFs subjected to *Meg3* knockdown (Supplementary Fig. S8A). Results from KEGG pathways analysis showed clustering of 1605 genes to only metabolic pathways (Supplementary Fig. S8B) and biological processes including metabolic terms such as positive regulation of metabolic processes, positive regulation of macromolecule metabolic processes, and positive regulation of nitrogen compound metabolic processes (Supplementary Fig. S8C). We next performed f-RIP with antibodies against H3K27ac, H3k27me3, and H3k9me3 from WT and β-actin KO condition. qPCR analysis with primers amplifying *Meg3* show that *Meg3* is specifically associated with H3K27ac and this interaction is enhanced in the β-actin KO condition. On the contrary *Meg3* binding to H3k27me3 and H3k9me3 is significantly reduced in the KO background (Fig. 6H). Based on these results, we hypothesize that *Meg3* binding at specific H3K27 acetylation sites close to or within gene regulatory regions is dependent on the nuclear actin pool.

Using HiC-Seq, ATAC-Seq, and RNA-Seq, we recently demonstrated that accessibility changes within compartment switching genes upon β-actin depletion are primarily observed in non-promoter regions such as distal regulatory elements [12]. The finding that β-actin loss induces widespread accumulation of H3K27ac [12] and that this enrichment happens at sites of *Meg3* enrichment suggest that *Meg3* may be involved in regulating transcription of certain metabolic genes at the level of promoter–enhancer interaction. Therefore, to find out if promoter–enhancer interactions are affected at *Meg3* enrichment sites in KO condition, we applied the ABC model of enhancer annotation based on HiC-Seq, ATAC-Seq, RNA-Seq, and H3K27ac ChIP-seq in WT and KO MEFs to study if these epigenetic changes have a direct impact on enhancer activity and underlie transcriptional changes. Based on the ABC results, PLD5 and PLCl1 gained an enhancer while HS3st3b1 lost an enhancer (Fig. 6I–K and Supplementary Table S3). Looking at the activity and contact components of ABC score which determines enhancer gain/loss, none of the genes show a major change in contact (see Fig. 6I and J). So, the gain/loss of enhancer identified in these genes is primarily driven by changes in activity, i.e. change in enhancer accessibility, particularly H3K27 acetylation (Fig. 6K). Interestingly, PLCL1 upregulation can be explained by increased enhancer Activity and HS3stb1 downregulation can be explained by decreased enhancer Activity (Fig. 6K). In contrast, PLD5 shows a very small increase in Activity so its downregulation cannot be entirely explained based on the ABC model. However, a close look at the integrated genomic visualization (IGV) shows that there is an enhancer that localizes within the promoter, and this is precisely where *Meg3* binding occurs concomitantly with increased H3K27ac in KO condition (Fig. 6C). So, PLD5 down regulation may be due to loss of both enhancer and promoter accessibility due to *Meg3* binding.

We, therefore, conclude that association of *Meg3* within or close to regulatory regions of the HS3ST3B1 and PLD5 genes impacts the promoter–enhancer interaction or promoter accessibility and leads to transcriptional inhibition. In contrast, *Meg3* enrichment does not affect regulation of the PLCL1 gene as its enrichment is far from the gene regulatory regions. These results also suggest that in the absence of β-actin increased *Meg3* binding is guided by local increase in H3K27ac level. In addition, *Meg3* primarily binds to or close to enhancers sites interfering with promoter–enhancer interaction or promoter accessibility required for gene activation, potentially contributing to alterations in the corresponding metabolic pathways.

## Discussion

In the cell nucleus β-actin plays essential functions in the 3D organization of the genome [12, 13]. We found that β-actin dependent changes in transcriptional and chromatin accessibility are highly enriched in compartment-switching regions. Furthermore, within compartment-switching genes, changes in chromatin accessibility were primarily found at promoter-distal regulatory elements [13, 25]. We also found that loss of nuclear β-actin leads to significant accumulation of the enhancer-specific epigenetic mark H3K27ac. Using the ABC model of enhancer annotation, we showed that H3K27ac accumulation affects enhancer activity and is an underlying cause of transcriptional changes during compartment switching. From these studies we concluded that enhancer-dependent transcriptional regulation plays a crucial role in driving gene expression changes upon compartment-switching. These observations are compatible with previous studies where actin was shown to control recruitment of both HDACs (histone deacetylases) and HATs (histone acetyltransferases) [14, 15]. Loss of β-actin disturbs the local epigenetic landscape and presumably impacts on promoter–enhancer interactions. The underlying mechanisms, however, on how promoter–enhancer interactions are affected in the absence of β-actin by increased H3K27ac remained unclear.

In this study, we show that upon β-actin depletion the levels of several lncRNAs including *Meg3* are significantly altered concomitantly with changes in H3K27 acetylation levels. H3K27ac plays an important role in the activation of several lncRNAs. For instance, CCAT1 and NEAT1 expressions are regulated by specific increase in H3K27 acetylation at their gene promoters [52, 53]. H3K27ac-dependent CCAT1 activation controls SPRY4 and HOXB13 expression during cell proliferation [52]. Increased H3K27ac levels at the NEAT promoter lead to increased NEAT expression, interaction with miR-212-5p followed by its suppression to regulate hepatic lipid accumulation [53]. Actin-dependent increase in H3K27ac levels at *Meg3* promoter and gene coding region also seem crucial for *Meg3* activation. This epigenetic landscape change is accompanied by increased chromatin accessibility in the β-actin KO condition (as revealed by ATAC-seq) and increased H3K27 acetylation levels.

Importantly, the general increase in *Meg3* levels in the KO condition has significant impact on global transcription at genomic level. ChIRP-seq using *Meg3*-specific probes showed that *Meg3* association with the genome changes in KO cells with respect to WT. Peaks of *Meg3* enrichment are observed at both promoter and non-promoter regions. They do not specifically correlate with chromatin compaction (see ATAC-seq data) or chromatin remodelers such as Brg1, Ezh2, and Suz12. There is, however, significant correlation between *Meg3* peaks in KO condition and increased levels of H3K27me3 and H3K27ac compared to WT. *Meg3* is known to functionally associate with the PRC2 complex and to facilitate histone methyl transferase activity. So, it is not surprising that upon β-actin depletion *Meg3* enrichment correlates with increased H3K27me3 levels given that PRC2 is recruited instead of the BAF complex [13]. On the other hand, the correlation between increased H3K27ac and *Meg3* in KO cells is less understood, but it might be involved in regulating gene promoter and its interaction with regulatory regions such as enhancers.

**Figure 7.**
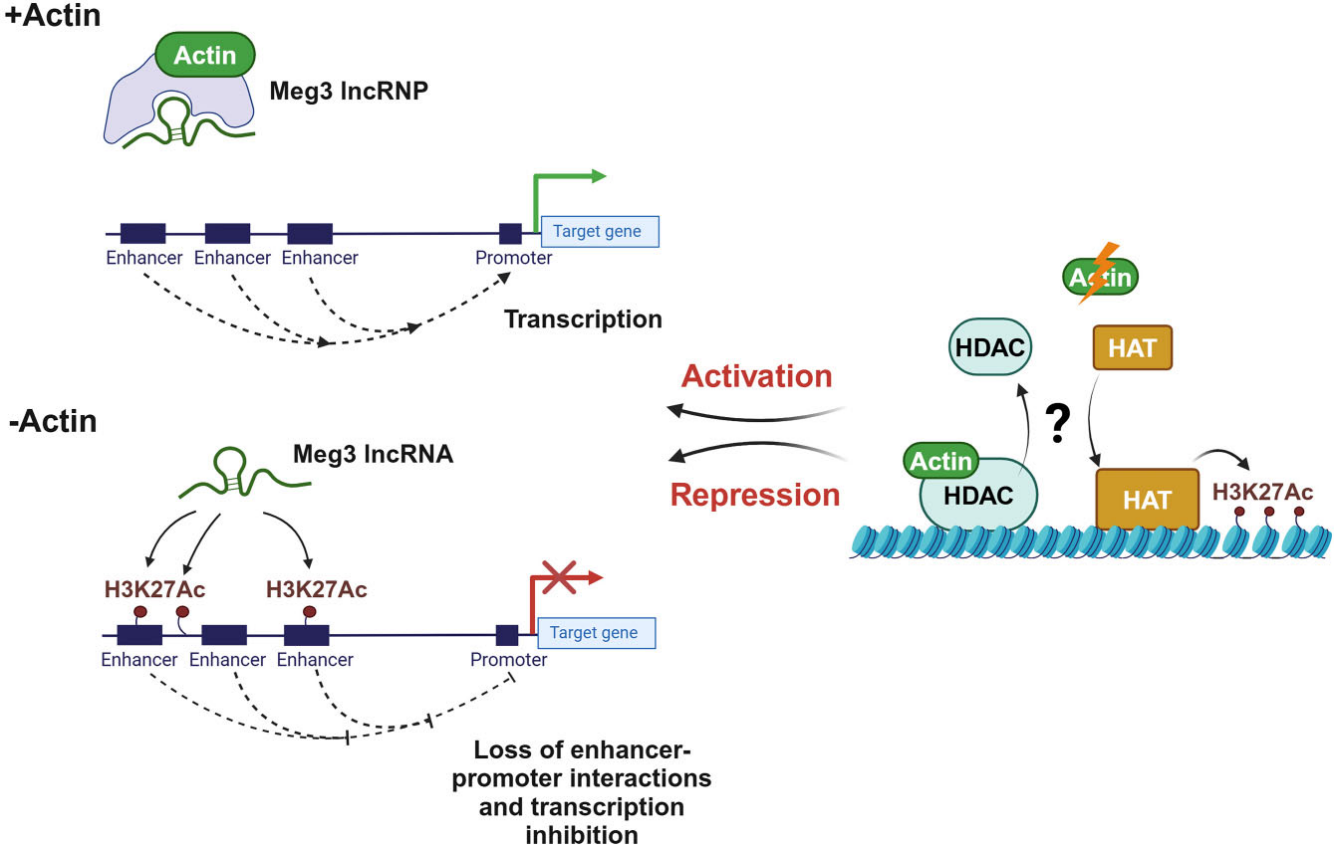
Proposed model showing the impact of β-actin on *Meg3* and its suggested role in genome organization. Upon β-actin depletion, the *Meg3* RNP is disassembled and *Meg3* binds to increased H3K27ac levels at gene regulatory regions and leads to transcription inhibition by interfering with promoter–enhancer interactions. We speculate that enhanced H3K27ac levels are a consequence of loss of HDACs recruitment that, in the absence of β-actin, is antagonized by HAT recruitment. Created in BioRender. Percipalle, P. (2025) https://BioRender.com/laojyaj

So, we started by exploring if in β-actin depleted cells *Meg3* binding at potential sites of H3K27 acetylation could be involved in controlling gene expression. In agreement with *Meg3* involvement in cell differentiation and metabolism [54], analysis of the RNAseq data in β-actin KO cells, showed that increased *Meg3* levels associate with differential expression of genes involved in metabolic pathways of chondroitin, heparan, dermatan sulfate, and phospholipases biosynthesis. Metabolomic profiling of WT and β-actin KO MEFs confirmed perturbations in chondroitin, heparan, and dermatan biosynthesis and phospholipases pathways in association with *Meg3* upregulation in the β-actin KO condition. Notably, *Meg3* is enriched at intergenic sites upstream the promoter of HS3ST3B1 (a gene that is central in the metabolic pathway leading to heparan biosynthesis). *Meg3* enrichment also peaked at an enhancer embedded within the gene promoter of the PLD5 gene (involved in phospholipase dependent metabolism). Both genes are downregulated and, in both cases, *Meg3* enrichment is at sites of increased H3K27ac levels. We next applied the ABC model for enhancer annotation to find out if *Meg3* could interfere with gene regulatory elements. We found that inhibition can be explained by a loss of activity-dependent (H3K27ac) promoter–enhancer interaction for HS3ST3B1 and, to a lesser extent, for the PLD5 gene. In contrast to HS3ST3B1 and PLD5, the PLCL1 gene is activated in the KO condition and *Meg3* binding seems to be lost from regulatory elements, including enhancers and promoter. f-RIP qPCR experiments show that *Meg3* directly binds to H3K27ac. Since knocking down Meg3 in the β-actin KO background rescues the levels of the HS3ST3B1, PLD5, and PLCL1 genes, we propose that upon β-actin *Meg3* plays a role in repressing gene expression by associating with regulatory elements, enhancers, and promoters, by interacting with H3K27acetylation (Fig. 7).

Compatible with the above model, ChIRP-MS results show that in the KO condition *Meg3* is primarily associated with histones in contrast to WT where *Meg3* binds β-actin and a set of hnRNP proteins. Early studies demonstrated that actin is a component of RNPs [19, 21]. In the 40S pre-mRNP/RNP fraction isolated from rat liver extracts [20], actin binds to core hnRNPs and these interactions are important for RNP assembly and transcription [17, 18, 21]. It is, therefore, possible that actin is a component of the *Meg3* RNP together with specific hnRNPs and that upon actin depletion the *Meg3* RNP is disassembled and *Meg3* binding to histones and thus, to the chromatin, is mediated by histone acetylation (see Fig. 7). We speculate that in the β-actin KO condition the lncRNA *Meg3* is not or only partially assembled into an RNP and this leads to *Meg3* collapse on the chromatin. We propose that *Meg3* binding to chromatin, is guided by enhanced H3K27ac levels. We speculate that this may be due to an actin-dependent loss of HDAC recruitment. So, serving as a decoy mechanism bound to the chromatin (referred to as sponging effect), we suggest that *Meg3* leads to a transcriptional block by affecting promoters and promoter–enhancer interactions, thus interfering with gene expression and key metabolic pathways.

## Supplementary Material

gkaf280_Supplemental_Files
