# Supplementary material for: Nuclear actin-dependent Meg3 expression suppresses metabolic genes by affecting the chromatin architecture at sites of elevated H3K27 acetylation levels
Source: Nucleic Acids Res. 2025 Apr 14;53(7):gkaf280. doi: 10.1093/nar/gkaf280 (PMC11995268; doi:10.1093/nar/gkaf280)

Supplementary figure legends

Supplementary figure 1. Genome-wide association of *Meg3* lncRNA is altered in the absence of β-actin. Merged replicates *Meg3* ChIRP-seq peaks coverage on WT (green) and NM1 KO MEFs (red) over mouse chromosomes, showing chromosome number and size.

Supplementary figure 2. ChIRP peaks annotation and elevated binding at TSS in KO. a) The Average Profile of ChIRP peaks binding to TSS region in WT and KO alone and upon b) overlapping, dark blue: KO; light blue: WT. c) heatmap of the *Meg3* ChIRP tags(reads), where more the tags fall in TSS we get redder shade. Heatmap of *Meg3* ChIRP peaks binding to TSS regions for WT and KO. d) UpSet plots for WT and e) KO datasets of the *Meg3* ChIRP, showing set data of *Meg3* ChIRP-seq over more than three intersecting sets. UpSet plots show intersections in a matrix, with the rows of the matrix corresponding to the sets, and the columns to the intersections between these sets. Genomic Regions, represent different regions of the genome that were bound by *Meg3* in the ChIRP-seq dataset. Each region corresponds to a specific genomic locus or set of loci. Intersection as connected dots represent the intersection of genomic regions and the Meg3 binding. It indicates the specific genomic regions where the captured *Meg3* -lncRNAs were found. The Venn diagrams within the Upset plot illustrate the overlap between different sets of genomic regions and *Meg3*. The bar shows the count of occurrences for each combination of sets. It provides a visual representation of the distribution of genomic regions and *Meg3* peaks across different subsets in WT and Actin KO MEFs.

Supplementary Figure 3. ChIRP peaks annotation shows *Meg3* preference in binding to specific genomic regions globally in KO vs WT. a) ChIRP-seq peak Annotation of WT-specific, KO-specific peaks and shared *Meg3* peaks in percentages of exon/intron, intergenic, Noncoding/TTS/, UTR and promoter/TSS. b) Boxplots showing average change in expression of genes overlapping or closest to (in case of intergenic peaks) *Meg3* peaks. Only genes showing differential expression with FDR <0.05 were included in the analysis. Scale bar shows normalized

RPKM. WT: RED, KO: Green, Shared peaks of WT and KO: blue. p-values based on two-tailed, two-sample Wilcoxon-rank sum test.

Supplementary Figure 4. ChIRP-seq shows *Meg3* bindings to and around TSS in KO specific, WT specific peaks and Shared *Meg3* peaks.

Density plots and heatmaps displaying scaled-read densities for *Meg3* in 3Kb regions centered on WT-specific, KO-specific, and shared *Meg3* Peaks. Expression is plotted as average log2FoldChange.

Supplementary figure 5. Illustrations of the dermatan sulfate (a), heparan sulfate (b) and Chondroitin sulfate (c) biosynthesis generated by Ingenuity Pathway Analysis (Qiagen) demonstrating the overrepresentation of genes implicated in these pathways Predicted gene and metabolite expression and activities are highlighted. Predictions are based on the fold change in gene expression levels between KO and WT replicates (n = 42,594 genes).

Supplementary Figure 6. Illustrations of the phospholipases pathways generated by Ingenuity Pathway Analysis (Qiagen) demonstrating the overrepresentation of genes implicated in these pathways. Predicted gene and metabolite expression and activities are highlighted. Predictions are based on the fold change in gene expression levels between KO and WT replicates (n = 42,594 genes).

Supplementary Figure 7. β-actin depletion affects accessibility at promoters of Metabolic genes. IGVs (integrated genomic visualization) of individual *Meg*3 ATAC-seq replicates for WT and β-actin KO conditions. Green: KO, Blue: WT. The reference gene body loci are shown at the bottom for Hs3st3b1 (a) Pld5 (b) and Plcl1(c). The y-axis represents RPKM (Reads Per Kilobase of sequence range per Million mapped reads) per bin. The range was set the same as in the image. shown below the tracks is the Gene body position for Hs3st3b1 (a) Pld5 (b) and Plcl1(c), (exon: box, intron: line).

Supplementary Figure 8. ***Meg3*** **depletion rescues the levels of Metabolic pathways genes in β-actin KO MEFs.** a) A PCA plot showing the differences and clustering of WT MEFS, KO MEFs and upon *Meg3* knockdown in WT MEFS +*Meg3* KD and KO MEFs + *Meg3* KD. b) KEGG pathways of genes enriched in β-actin KO MEFs + *Meg3* KD showing only 1605 genes involved significantly in metabolic pathways only. c) GO analysis of biological processes (BP) of the genes enriched in the KO MEFs + *Meg3* KD.

Supplementary figure 1

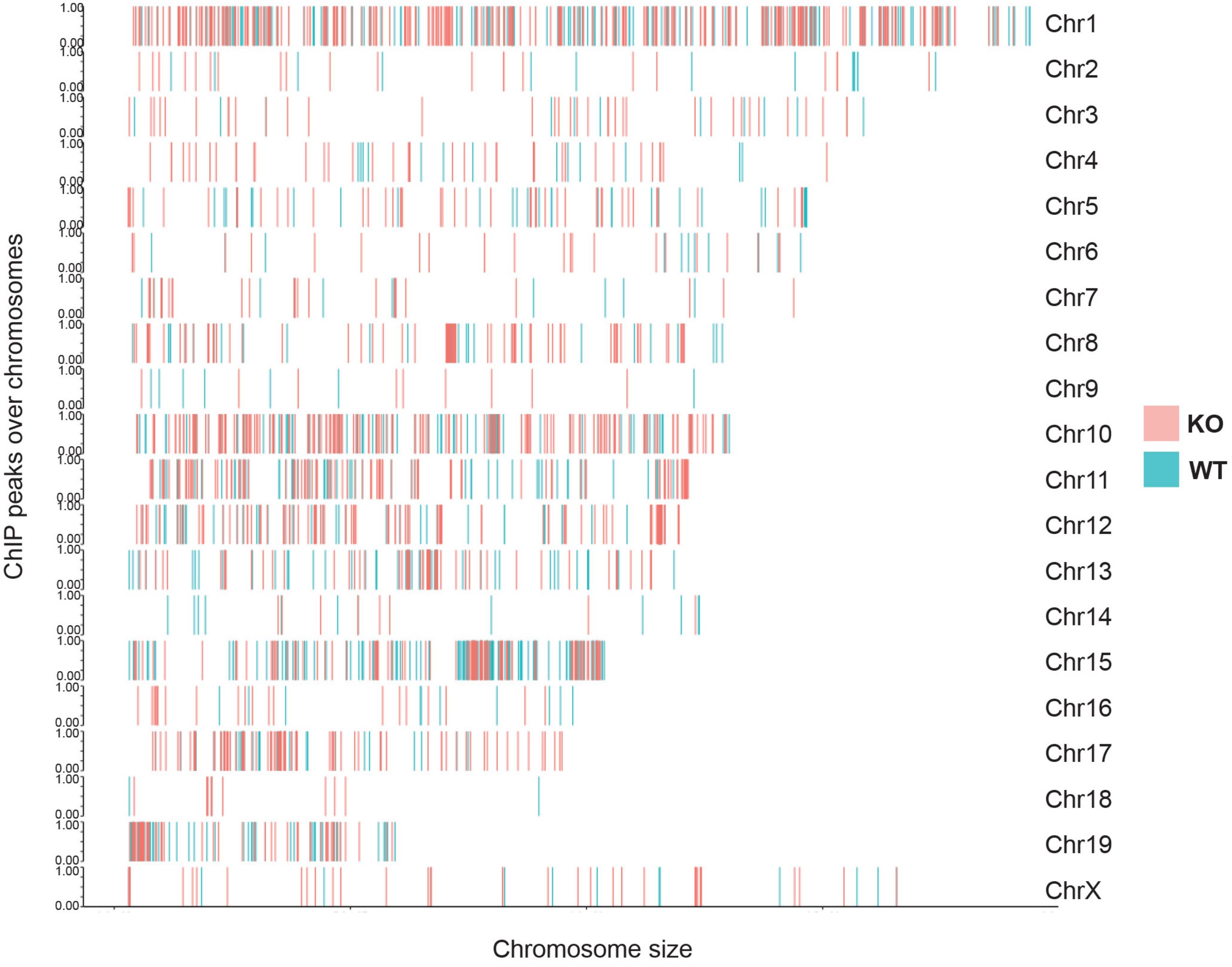

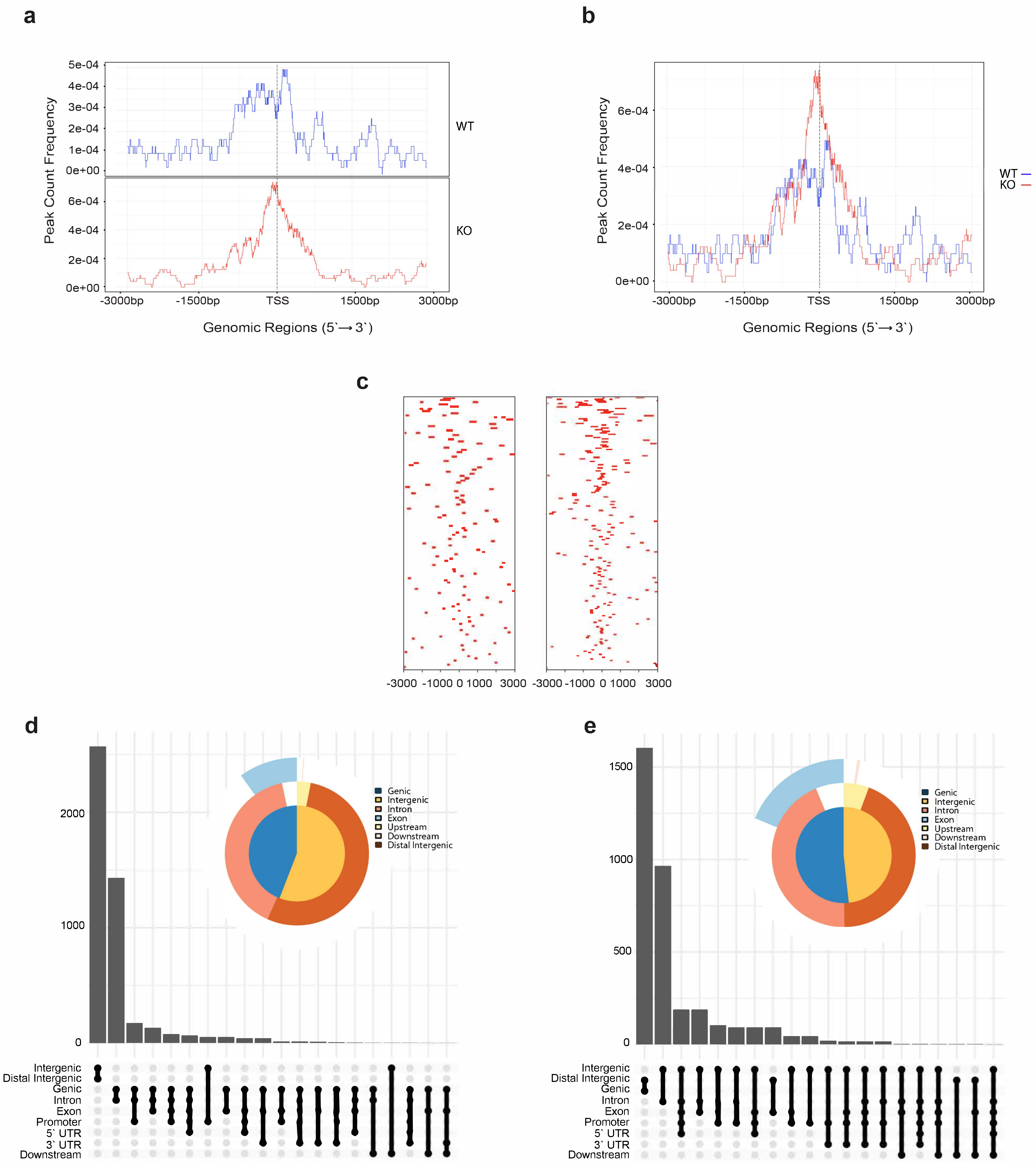
a
b
Peak Count Frequency
WT
KO
TSS
-3000bp
-1500bp
1500bp
3000bp
Genomic Regions (5`⟶3`)
c
-3000 -1000 0 1000 3000
d
e
Genic
Intergenic
Intron
Exon
Upstream
Downstream
Distal Intergenic
Promoter
5` UTR
3` UTR

Supplementary figure 3

a

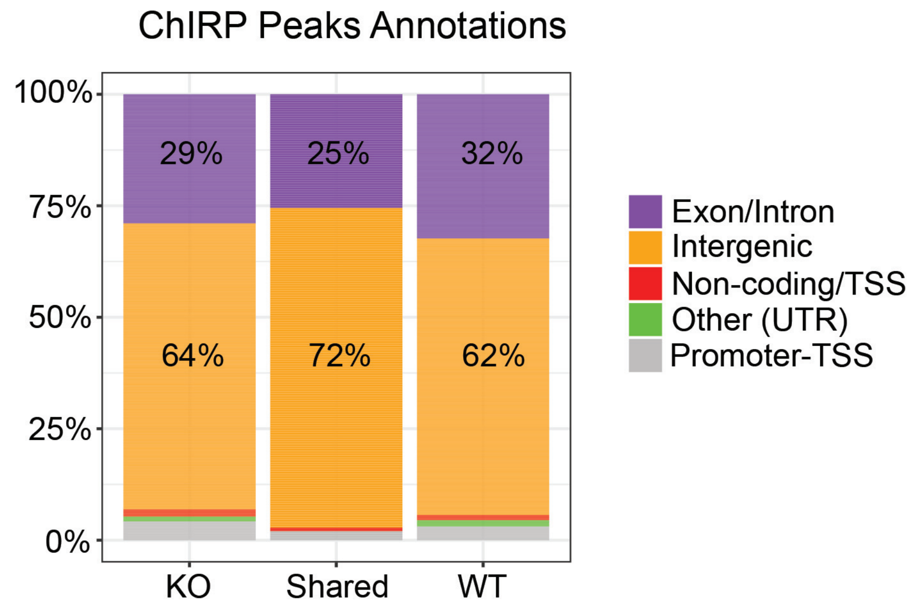

b

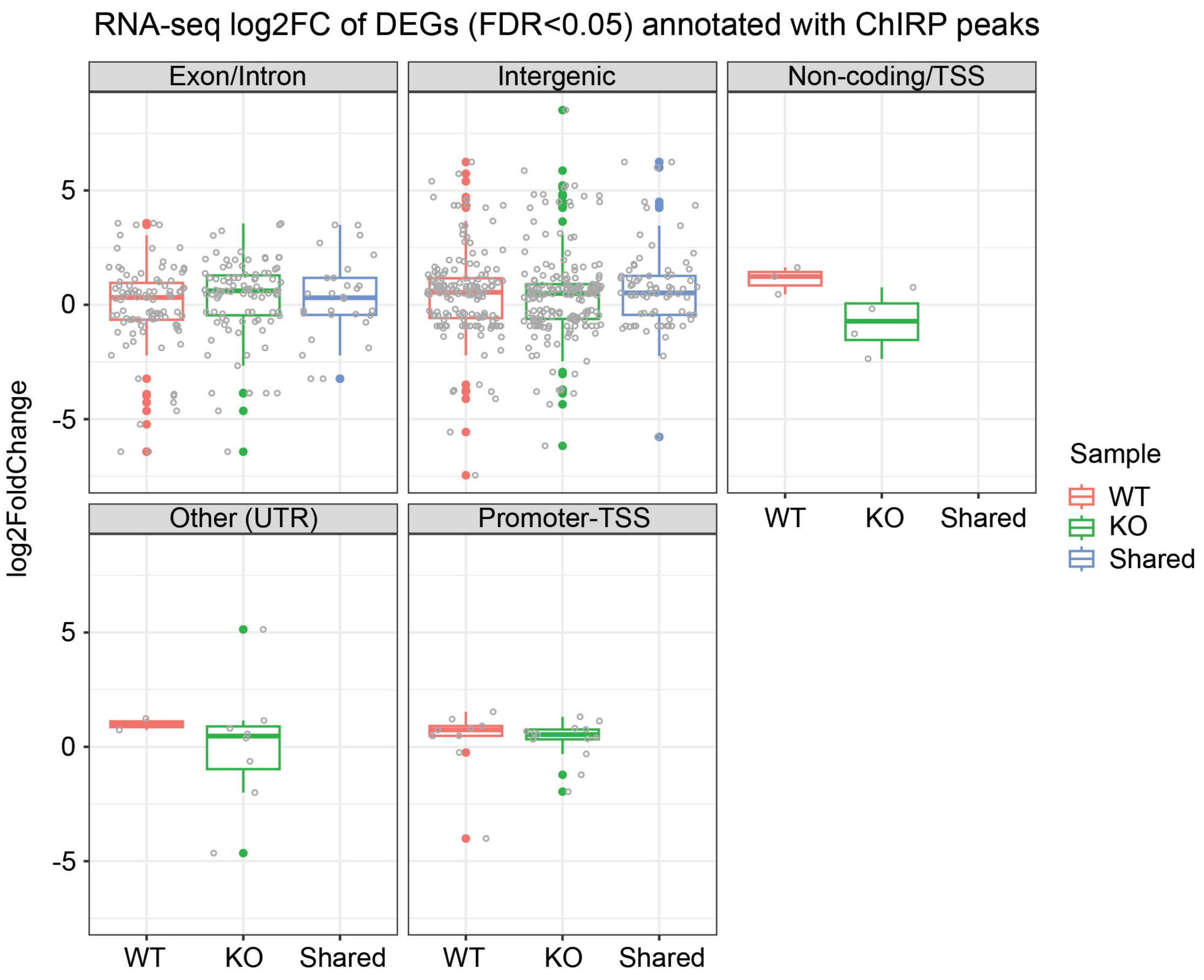

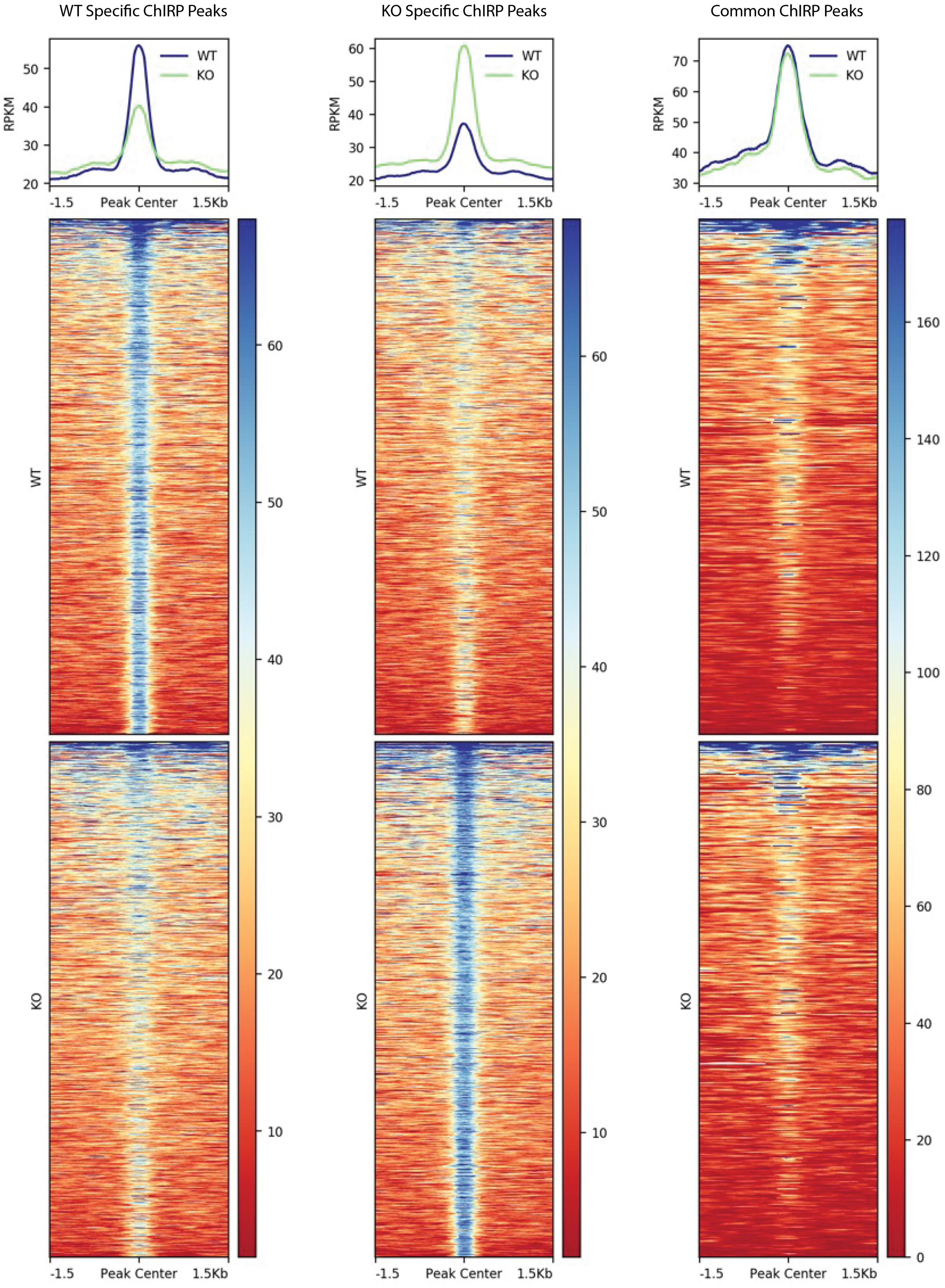
WT Specific ChIRP Peaks
KO Specific ChIRP Peaks
Common ChIRP Peaks
RPKM
WT
KO
-1.5
Peak Center
1.5Kb
WT
KO

Supplementary figure 5

a

Dermatan sulfate biosynthesis pathway

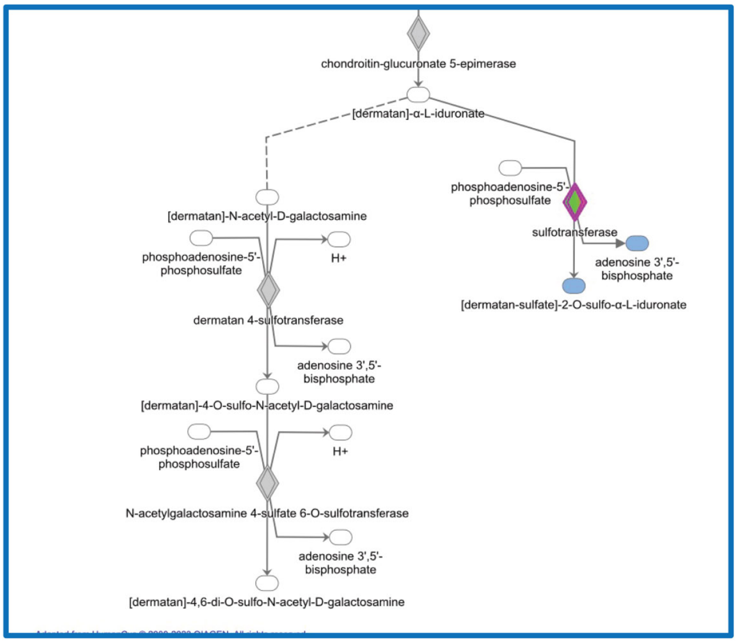

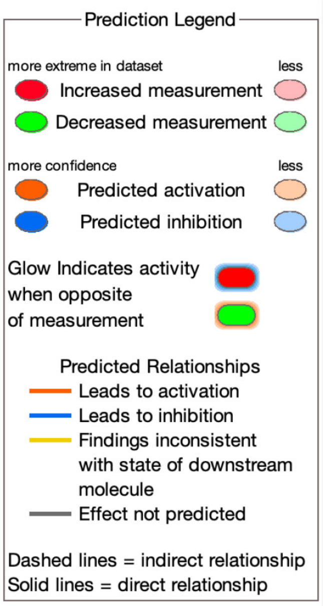

b

Heparan sulfate biosynthesis pathway

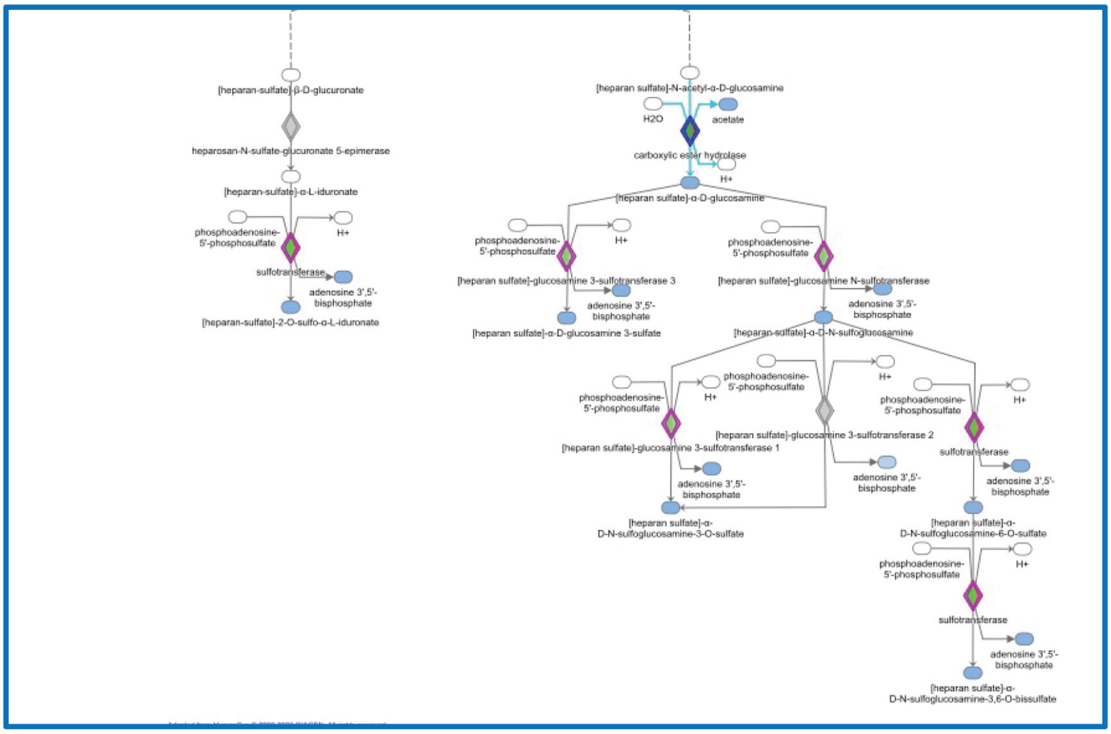

c

Chondroitin sulfate biosynthesis pathway

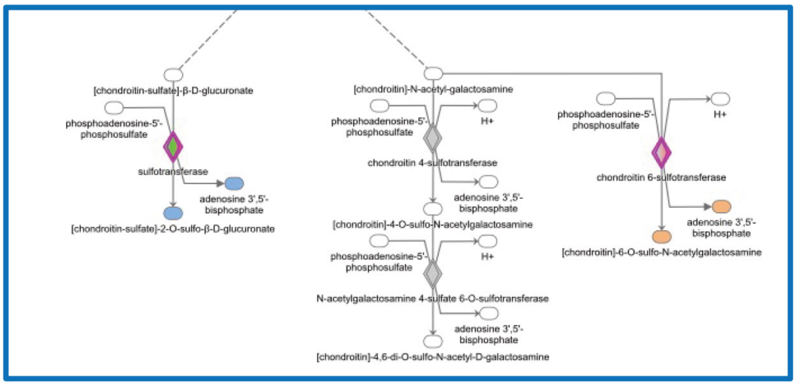

Supplementary figure 6

Phospholipases biosynthesis pathways

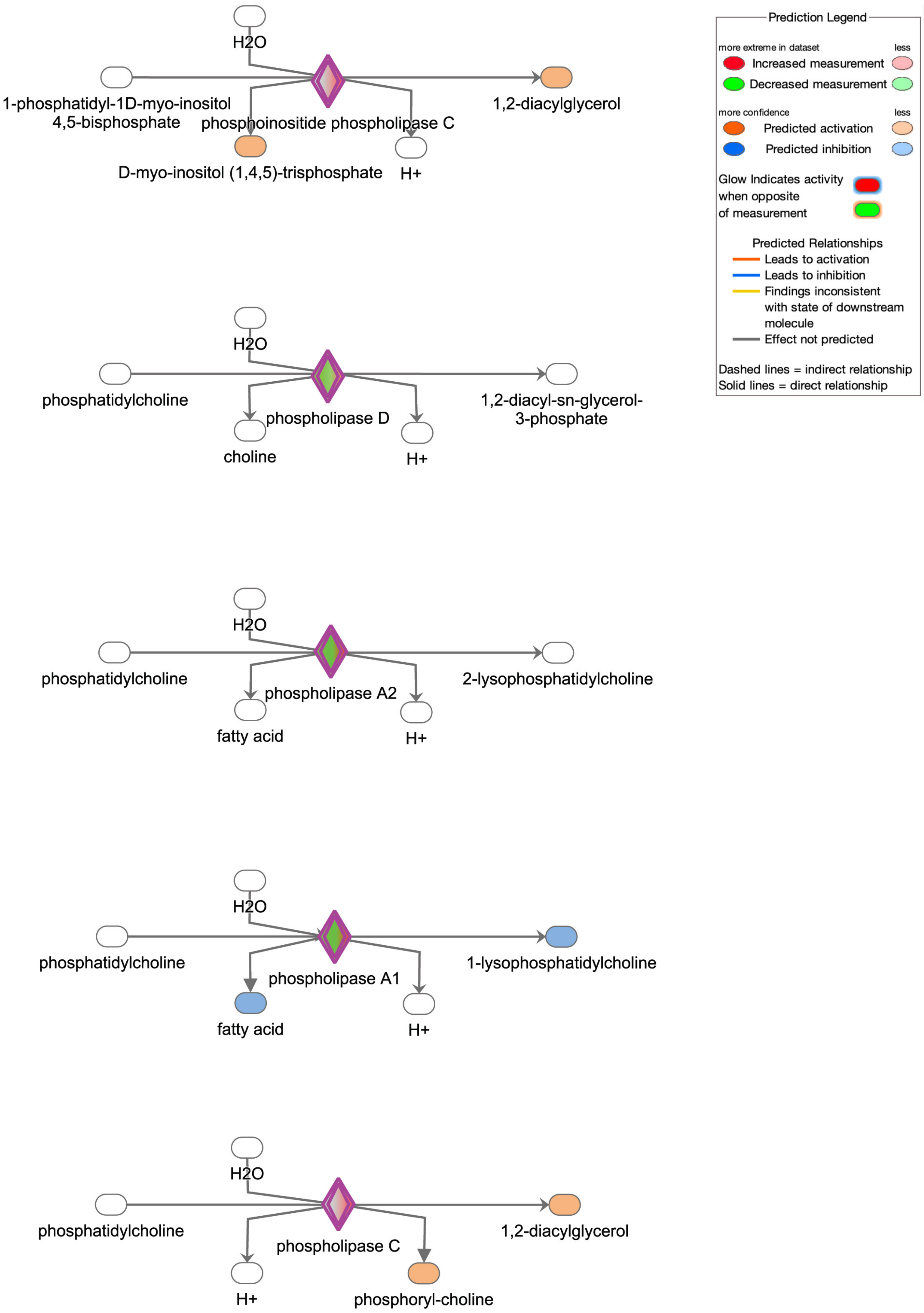

Supplementary figure 7

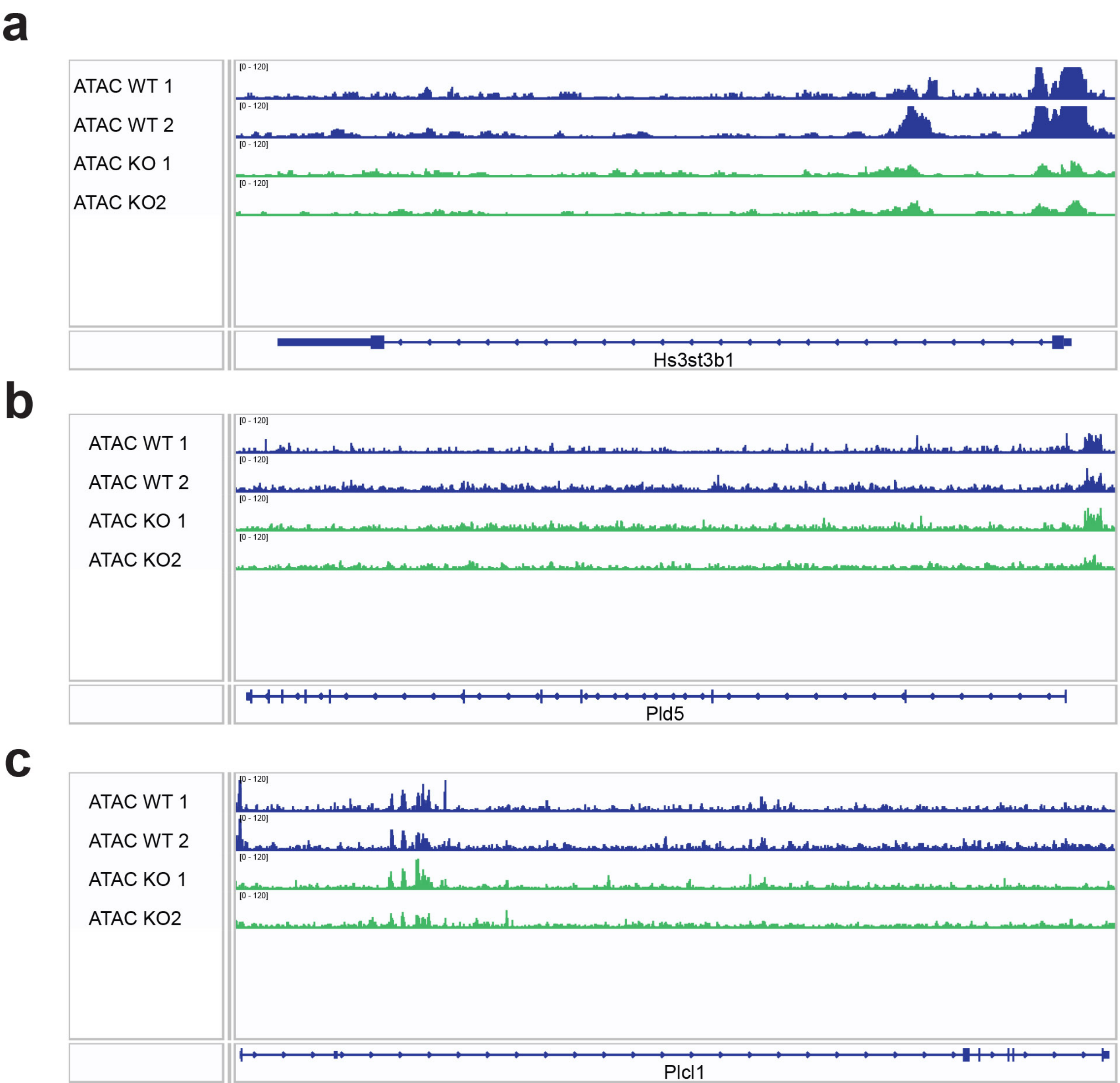

Supplementary figure 8

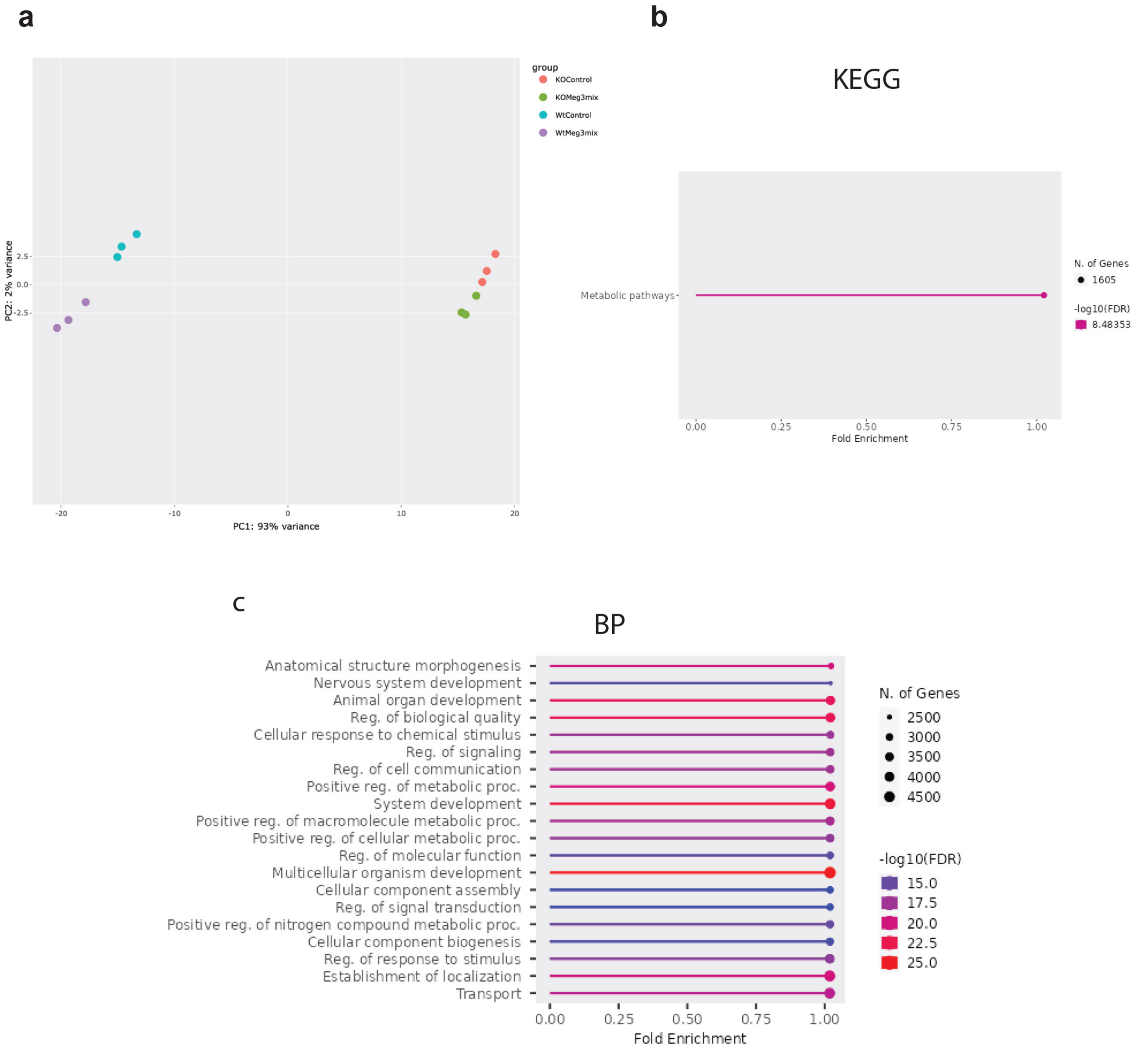

Supplement: gkaf280_Supplemental_Files [file gkaf280_supplemental_files.zip › El Said et al 2024 - supplementary figures and figure legends - revised.pdf]
